# Improving Hospital Oxygen Systems for COVID-19 in Low-Resource Settings: Lessons From the Field

**DOI:** 10.9745/GHSP-D-20-00224

**Published:** 2020-12-23

**Authors:** Hamish R. Graham, Sheillah M. Bagayana, Ayobami A. Bakare, Bernard O. Olayo, Stefan S. Peterson, Trevor Duke, Adegoke G. Falade

**Affiliations:** a Centre for International Child Health, University of Melbourne, MCRI, Royal Children’s Hospital, Melbourne, Australia.; b Department of Paediatrics, University College Hospital, Ibadan, Oyo, Nigeria.; c FREO2 Uganda, FREO2 Foundation, Kampala, Uganda.; d Biomedical consultant, Uganda Ministry of Health, Kampala, Uganda.; e Department of Community Medicine, University College Hospital, Ibadan, Oyo, Nigeria.; f Oxygen for Life Initiative, Oyo, Nigeria.; g Center for Public Health and Development, Nairobi, Kenya.; h Chief of Health, United Nations Children’s Fund, New York, NY, USA.; i Department of Women's and Children's Health, Uppsala University, Uppsala, Sweden.; j Department of Global Public Health, Karolinska Institutet, Stockholm, Sweden.; k School of Public Health, Makerere University, Kampala, Uganda.; l School of Medicine and Health Sciences, University of Papua New Guinea, National Capital District, Papua New Guinea.

## Abstract

Oxygen therapy is an essential medicine and core component of effective hospital systems. However, many hospitals in low- and middle-income countries lack reliable oxygen access—a deficiency highlighted and exacerbated by the coronavirus disease (COVID-19) pandemic. Oxygen access can be challenged by equipment that is low quality and poorly maintained, lack of clinical and technical training and protocols, and deficiencies in local infrastructure and policy environment. We share learnings from 2 decades of oxygen systems work with hospitals in Africa and the Asia-Pacific regions, highlighting practical actions that hospitals can take to immediately expand oxygen access. These include strategies to: (1) improve pulse oximetry and oxygen use, (2) support biomedical engineers to optimize existing oxygen supplies, and (3) expand on existing oxygen systems with robust equipment and smart design. We make all our resources freely available for use and local adaptation.

## Abstract (precis)

Hospitals in low- and middle-income countries urgently need to improve their oxygen systems for COVID-19 and other health emergencies. We share practical tips to improve pulse oximetry and oxygen use, support biomedical engineers to optimize existing oxygen supplies, and expand existing oxygen systems with robust equipment and smart design.

## Teaser

**Key Messages:**

- The COVID-19 pandemic has highlighted and exacerbated deficiencies in hospital oxygen systems globally but is also an opportunity to “build back better.”
- Our collated field experience from African and Asia-Pacific contexts reveal practical strategies whereby hospitals can rapidly improve their oxygen systems. We share guidance documents (all open access) for local use and adaptation.

**Key Implications:**

- Using our practical guides, hospital staff can help:
  - Improve pulse oximetry and oxygen use
  - Optimize existing oxygen supplies
  - Expand existing oxygen systems with robust equipment and smart design
- Policy makers and program managers can use our recommendations to ensure that investments in oxygen systems are deployed and implemented more effectively and efficiently.

## BACKGROUND

Oxygen therapy is an essential medicine and core component of hospital systems that has been a standard of care for more than 100 years.^1^ However, access to oxygen therapy is limited in many low-resource settings, where the majority of hypoxemic patients who are admitted to the hospital will not receive oxygen, resulting in an increased risk of death.^2^

The coronavirus diseases (COVID-19) pandemic has revealed the extent of this “oxygen gap” and stimulated long overdue interest in improving oxygen systems. Approximately 20% of patients who have COVID-19 require hospital admission for oxygen therapy (with or without extra respiratory support).^3^ Although much attention has focused on ventilator and intensive care unit capacity, improving basic hospital oxygen systems must take priority.^4^

## CHALLENGES IN OXYGEN ACCESS

To provide oxygen therapy, we need a reliable oxygen supply, prompt identification of patients requiring oxygen therapy, and appropriate administration by skilled health care workers.^5^ Oxygen supply is typically achieved using oxygen cylinders (filled at an oxygen plant), oxygen concentrators (concentrating oxygen from air on-site), oxygen plants (piped directly or distributed via cylinders), or liquid oxygen (delivered from a specialized gas plant and stored on-site at very high pressure). Oxygen use is guided by nurses measuring blood oxygen levels using a pulse oximeter (or relying on clinical signs if no oximeter is available) and titrating oxygen flow rate to maintain adequate blood oxygen levels.

However, achieving reliable supply and appropriate use is challenging, with major barriers due to equipment that is low quality and poorly maintained, lack of clinical and technical training and protocols, and deficiencies in local infrastructure and sociopolitical context.^6^

Achieving reliable oxygen supply and appropriate use is challenging, with barriers due to low-quality and poorly maintained equipment and lack of clinical and technical training and protocols.

For example, surveys in Nigeria have found that although half of hospitals had oxygen cylinders or concentrators on inpatient wards, the cylinders and concentrators were frequently empty or nonfunctional.^2^ ^,^ ^7^ Detailed testing in a selection of these hospitals found that only 5% of concentrators tested were producing medical grade oxygen.^2^ Almost no hospitals had pulse oximeters available on the wards.^2^ ^,^ ^7^ Procurement of oxygen equipment was haphazard, preventive maintenance was nonexistent, and hospital technicians were untrained and under-supported. Hospital nurses were unfamiliar with pulse oximetry, and the majority of hypoxemic patients were not receiving oxygen.^2^ ^,^ ^7^ Hospital directors bemoaned the cost of oxygen, with one director describing oxygen as his “biggest headache.”

We, and others, have reported similar findings in Kenya, Uganda, Papua New Guinea, and other African and Asia-Pacific contexts.^7^ ^–^ ^11^ Indeed, unreliable oxygen supplies and deficiencies in oxygen use are consistent and persisting problems for many hospitals in these regions, particularly in rural and remote settings.

Challenges to oxygen access exist alongside broader systems issues such as unreliable power supply, health care workforce constraints, high out-of-pocket health care costs, low health literacy, and underfunded public health and preventive services.

However, our work has shown that effective and sustainable change is possible.

## LESSONS LEARNED

Over the past 2 decades, we have supported hospitals in Africa and Asia-Pacific regions to improve oxygen systems using low-cost technology such as oxygen concentrators, pulse oximeters, and cylinder distribution systems. Our work has shown how to combine quality equipment and training to achieve context-appropriate and sustainable improvement in oxygen systems and improve clinical outcomes.^5^ ^,^ ^11^ ^–^ ^13^ This experience has informed the development of clinical and technical guidance by the World Health Organization (WHO) and the United Nations Children’s Fund (UNICEF)^14^ ^–^ ^16^ and is now informing oxygen scale-up for COVID-19 (Box).

BOX Essential Resources for Additional Information on Oxygen and COVID-19

- Repository of oxygen resources (curated by the United Nations Children’s Fund [UNICEF]): https://bit.ly/OxygenResources
- World Health Organization (WHO) COVID-19 Technical Guidance: Essential Resource Planning: https://www.who.int/emergencies/diseases/novel-coronavirus-2019/technical-guidance/covid-19-critical-items
- WHO COVID-19 Technical Guidance: Patient Management: https://www.who.int/emergencies/diseases/novel-coronavirus-2019/technical-guidance/patient-management
- WHO Academy’s COVID-19 mobile learning app for Android and iPhone/iPad, which contains much of the clinical and technical advice in a conveniently accessible format.
- WHO COVID-19 Partners Platform: https://covid-19-response.org/, which includes a supply portal for requesting and receiving critical supplies.
- WHO Medical Devices for COVID-19: https://www.who.int/medical_devices/priority/COVID-19_medequipment/en/
- UNICEF supply catalogue: https://supply.unicef.org/. Order via UNICEF Country Office.
- UNICEF Supply Division COVID-19 response: https://www.unicef.org/supply/coronavirus-disease-covid-19

We show how to combine quality equipment and training to achieve sustainable improvement in oxygen systems and improve clinical outcomes.

To complement existing technical guidance on COVID-19 response from WHO^17^ and others, we offer practical suggestions based on our on-the-ground experience to help policy makers, administrators, technicians, and health care workers seeking to rapidly improve their hospital oxygen systems.

### 1. Support Health Care Workers to Use Pulse Oximetry and Oxygen Through Training and Protocols

In many low- and middle-income countries (LMICs), oxygen is absent from medical and nursing training,^18^ and pulse oximetry is unavailable.^2^ Pulse oximetry, with practical task-based training and simple guidelines, can enable health care workers to target oxygen toward those who need it most, dramatically improving patient oxygen access and clinical outcomes.^13^ In contexts where pulse oximetry is not a standard of care, COVID-19 offers an opportunity to establish oxygen saturation as the “fifth vital sign.” However, although pulse oximetry is a simple skill, it is a fundamentally new concept for many health care workers and will require encouragement and support to integrate it into the workflow.^19^ Education and support for health care workers to use pulse oximetry and oxygen therapy should also cover maintenance and functioning of oxygen equipment, recognizing the critical importance of health care worker and technician teamwork in maintaining a reliable oxygen supply. We have created practical training materials, clinical algorithms, and troubleshooting guides, based on WHO guidelines, for others to use and adapt (Supplements 1–3).

**Figure:**
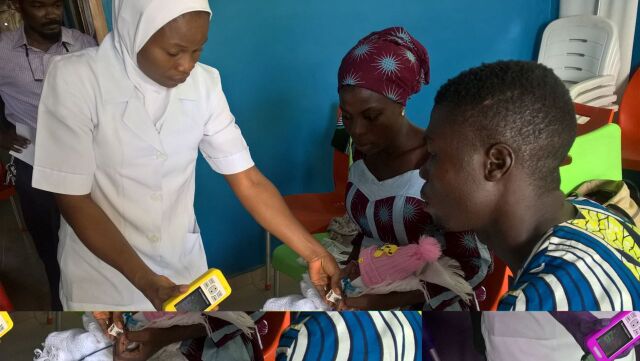
Nurse in Nigeria performing pulse oximetry on an infant, demonstrating oxygen saturation level to parents. © 2017 Oxygen for Life Initiative

Pulse oximetry can enable health care workers to target oxygen to those who need it, dramatically improving oxygen access and clinical outcomes.

### 2. Assist Biomedical Engineers to Optimize Existing Oxygen Supplies Through Training, Protocols, and Logistic Support

Oxygen is a medicine that depends on technology and requires effective teamwork between health care workers, technicians, and managers. However, biomedical engineers and hospital technicians are frequently left out of decision-making processes and lack maintenance budgets or system support. Training, provision of tools and spare parts, and stronger maintenance and transport systems can enable repair and optimization of existing oxygen equipment and supply chains. Installation of simple piping and individual flowmeters can improve safety (allowing individual titration of flow), efficiency (sharing a single oxygen source between multiple patients) and infection control (allowing oxygen sources to be kept away from patient areas). Including technicians alongside health care workers in multidisciplinary teams can help transform a problem-driven “oxygen headache” into focused oxygen solutions. We have created practical resources to assist biomedical engineers/technicians to build and maintain reliable, user-friendly oxygen systems using oxygen concentrators and/or cylinders, flowmeter stands, and simple piping (Supplement 4).

**Figure:**
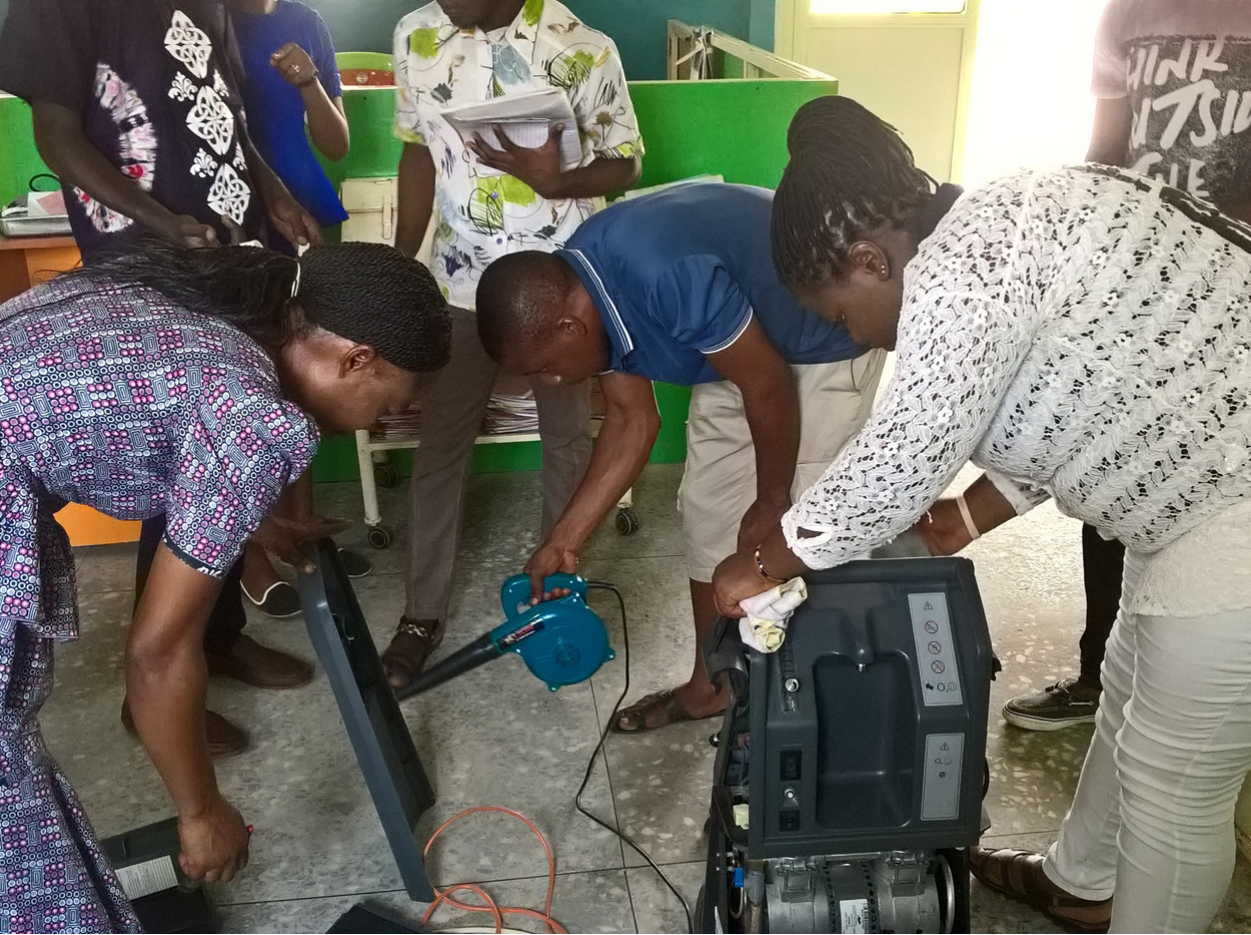
Technicians in Nigeria learning to perform preventive maintenance and repair an oxygen concentrator. © 2017 Oxygen for Life Initiative

### 3. Expand on Existing Oxygen Systems Using Robust Equipment and Smart Design

WHO and UNICEF have released guidance on oxygen-related equipment^14^ ^,^ ^16^ and specific guidance for COVID-19.^17^ This guidance includes low-cost oxygen concentrator-based systems that use simple plastic piping and flowmeter stands to provide oxygen to multiple patients simultaneously. These systems have been successfully implemented in African and Asia-Pacific contexts and can be established in a relatively short time frame (compared to a new oxygen plant). With the support of several other donors, UNICEF has delivered almost 15,000 oxygen concentrators and approximately 15,000 pulse oximeters to 69 countries (at the time of writing). Many other donors have channeled equipment support directly. However, there is a real risk that these valuable investments will end up in equipment graveyards with inadequate consideration to how they are deployed in hospitals. Hospitals can use our practical installation guidance to create smart and efficient ward oxygen systems to put this influx of equipment to use rapidly and effectively (Supplement 4).

**Figure:**
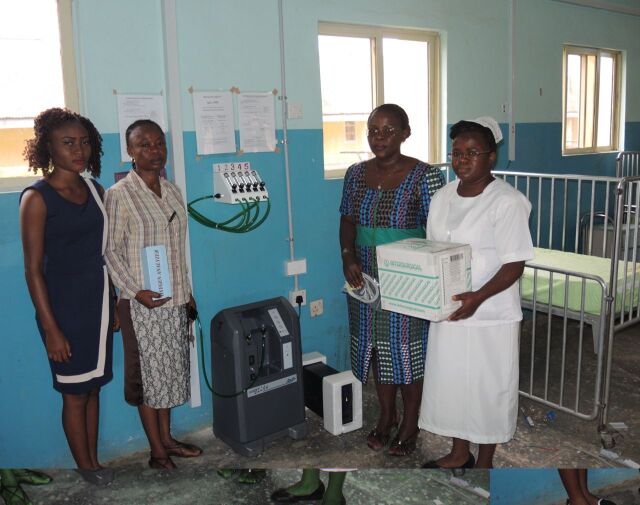
Technician, doctor, and nurse in Ondo state, Nigeria, with new oxygen installation including oxygen concentrator with power stabilizer, oximeter, flowmeter stand, distribution tubing, oxygen analyzer, and user guides. © 2016 Oxygen for Life Initiative

Hospitals can use our practical installation guidance to create smart and efficient ward oxygen systems to put this influx of equipment to use rapidly and effectively.

## CONCLUSIONS

Improving patient outcomes always hinges on doing the basics well. The COVID-19 pandemic offers the opportunity to refocus efforts on the basics of acute care, knowing that improvements in oxygen (as well as infection control, triage, laboratory testing, etc.) will benefit patients both now and in the future.

Improving oxygen systems is an achievable priority for hospitals in LMICs. We propose practical steps to support effective and sustainable improvements in hospital oxygen systems during the COVID-19 pandemic. We share these learnings in the hope that health care workers, technicians, hospital managers, and policy makers will be able to take immediate actions toward better oxygen access. All the accompanying oxygen resources are freely available for users to download, use, and adapt to local needs (https://bit.ly/OxygenResources). We welcome your feedback.

## Supplementary Material

20-00224-Graham-Supplement3.pdf

20-00224-Graham-Supplement2.pdf

20-00224-Graham-Supplement1.pdf

20-00224-Graham-Supplement4.pdf

## Notes

**Peer Reviewed**

**First published online:** September 28, 2020
