# Supplementary material for: Improving Hospital Oxygen Systems for COVID-19 in Low-Resource Settings: Lessons From the Field
Source: Glob Health Sci Pract. 2020 Dec 23;8(4):858–62. doi: 10.9745/GHSP-D-20-00224 (PMC7784072; doi:10.9745/GHSP-D-20-00224)
Supplement: 20-00224-Graham-Supplement2.pdf [file 20-00224-Graham-Supplement2.pdf]

Supplement to: Graham HR, Bagayana SM, Bakare AA, et al. Improving hospital oxygen systems for COVID-19 in low-resource settings: lessons from the field. Glob Health Sci Pract. 2020;8(4). https://doi.org/10.9745/GHSP-D-20-00224

DOES THIS PATIENT NEED OXYGEN?

Is this an Emergency?

Airway / Breathing: Obstructed or absent breathing, Central cyanosis, Severe respiratory distress
Circulation: Capillary refill >3 seconds, Weak and fast pulse
Disability: Coma, Convulsing (seizures)

YES →

Give oxygen during RESUSCITATION regardless of SpO_2

After resuscitation → Check blood oxygen saturation (SpO_2) with pulse oximeter*

NO ↓

Check blood oxygen saturation (SpO_2) with pulse oximeter*

$SpO_2 \geq 90\%$ → Continue clinical assessment and care

$SpO_2 < 90\%$ ↓

Give oxygen using nasal prongs

Child: 0.5-2L/min via nasal prongs or catheter
Adult: 2-4L/min via nasal prongs or mask

Target SpO_2 >90%
Check after 15 minutes

In special cases, consider giving O_2 to keep SpO_2 ≥94% and protect the brain (e.g. head injury, severe anaemia, heart failure, sepsis, peri-operatively).

SpO_2 ≥90% → Continue clinical assessment and care

$SpO_2 < 90\%$ ↓

Increase low-flow oxygen to maximum

Child: 2-4L/min via nasal prongs
Adult: 5-15L/min via non-rebreather mask

Check after 15 minutes

SpO_2 ≥90% → Continue clinical assessment and care

$SpO_2 < 90\%$ ↓

Admit to intensive-care, if available

Maximise oxygen support and consider escalating respiratory support if available (e.g. CPAP, high flow).
Monitor SpO_2 continuously, if possible.

Continue clinical assessment and care

Check SpO2 and vital signs every 4-6 hours while on oxygen and according to illness severity.

Maintain SpO2≥90%, reducing oxygen flow rate when possible.

Check SpO_2 immediately if patient develops: Apnoea; Respiratory distress; or Altered conscious state

---

* If you do not have a pulse oximeter, use clinical signs:

central cyanosis, severe lower chest wall indrawing, RR≥70 breaths per minute, inability to drink (due to respiratory distress), grunting with every breath, depressed mental state.

OXYGEN FOR LIFE https://oxygen4life.org/ Updated 30 March 2020

HOW TO PERFORM (AND TROUBLESHOOT) PULSE OXIMETRY

Prepare oximeter and patient

- Select appropriate probe for patient
- Connect probe to oximeter and turn oximeter on
- Tell the patient/parent what you are going to do
- Position the patient so they are comfortable (e.g. on parent's lap)

Attach probe to patient and wait

- Attach probe to a finger, toe, or ear
- Wait until there is a regular pulse signal (may take 20-30 seconds)

Is there a good Pulse Signal (waveform)?

NO → Troubleshoot (then recheck)

Troubleshoot

- Reposition probe
- Check circulation (if poor, resuscitate immediately)
- Check oximeter function on own finger
- Warm the finger or toe

recheck

YES ↓

Read the SpO2 - record SpO2 on a monitoring chart

What is the SpO_2?

≥90% → Continue clinical assessment and care

<90% → Treat immediately

Continue clinical assessment and care

Check SpO2 and vital signs every 4-6 hours while on oxygen and according to illness severity.

Maintain SpO2≥90%, reducing oxygen flow rate when possible.

Check SpO_2 immediately if patient develops: Apnoea; Respiratory distress; or Altered conscious state

Clean the nasal prongs each time you check the SpO2 to keep them patent

----

In <u>Emergencies</u> give oxygen during resuscitation regardless of SpO_2 : obstructed/absent breathing, central cyanosis, severe respiratory distress, capillary refill >3 seconds, weak & fast pulse, coma, seizures.

In <u>Special Cases</u> give oxygen to keep SpO_2 ≥94% and protect the brain: head injury, meningitis, severe anaemia, heart failure, sepsis, peri-operatively.

Treat <u>immediately</u> if SpO_2 <90%

(assume HYPOXIA until proven otherwise)

- Give oxygen: Child = 0.5-2L/min via nasal prongs or catheter; Adult = 2-4L/min via nasal prongs or mask
- Recheck SpO_2: record SpO_2, O_2 flow rate

Recheck the SpO_2?

≥90% → Continue clinical assessment and care

<90% → Increase O_2 therapy and recheck patient

Increase O_2 therapy and recheck patient

(it is essential to correct hypoxia)

Turn oxygen flow rate up to increase SpO_2 ≥90%

- Maximum rate: 4L/min for a child, 2L/min for an infant, 5-15L/min for an adult (non-rebreather mask).

If remains low

- Check equipment (oximeter and oxygen supply)
- Check patient airway, suction nose
- Check circulation
- Consider medical causes (e.g. pneumothorax, congenital heart disease)
- Consider extra respiratory support (e.g. CPAP)

MONITORING AND STOPPING OXYGEN THERAPY

Adults and Children (except preterm/small neonates)

Check blood oxygen saturation (SpO_2) using pulse oximetry

- Attach probe to a finger, toe, or ear
- Wait until there is a regular pulse signal (this may take 60 seconds)

Troubleshoot

- Reposition probe
- Check circulation (if poor, resuscitate immediately)
- Check oximeter function on own finger
- Warm the finger or toe

Read the SpO_2 - record SpO_2 and oxygen flowrate on a monitoring chart

What is the SpO_2?

SpO_2 <90%

TOO LOW => <u>Increase</u> O_2 therapy

Max flow-rate:
Child = 2-4L/min via nasal prongs
Adult = 5-15L/min via non-rebreather mask

Check the SpO_2 after 15 minutes, repeat in an hour

SpO_2 ≥90% (→ GOOD)

SpO_2 <90%

Consider increasing respiratory support

Admit to intensive-care.

Maximise oxygen support and consider escalating respiratory support if available (e.g. high flow, CPAP, NIV/BiPAP).

Monitor SpO_2 continuously.

SpO_2 ≥90%

GOOD => <u>Continue</u> O_2 therapy

Continue routine monitoring

If <u>SpO_2 ≥96%</u>, try reducing (or ceasing) oxygen therapy daily

Check the SpO_2 after 15 minutes, repeat in an hour

Repeat as above

Routine SpO_2 monitoring

Monitor SpO_2 and vital signs at least 4 times daily in all patients, and 4-6 hourly while on oxygen

- Aim to keep SpO_2 between 90-95% at all times
- Monitor more frequently in patients with severe respiratory distress (preferably continuously)
- Recheck SpO_2 immediately if patient develops apnoea, worsening respiratory distress, altered conscious state, or other signs of clinical deterioration
- Consider oxygen therapy to maintain SpO_2 ≥95% in special cases (e.g. severe anaemia, shock)

Document SpO_2 and oxygen flow rate on a monitoring chart
Clean the nasal prongs each time you check the SpO_2 to ensure they are patent

MONITORING AND STOPPING OXYGEN THERAPY

Preterm (<37 weeks GA), Small (birth weight <2000g)

Check blood oxygen saturation (SpO_2) using pulse oximetry

- Attach probe to a finger, toe, or ear
- Wait until there is a regular pulse signal (this may take 60 seconds)

Troubleshoot

- Reposition probe
- Check circulation (if poor, resuscitate immediately)
- Check oximeter function on own finger
- Warm the finger or toe

Read the SpO_2 - record SpO_2 and oxygen flowrate on a monitoring chart

What is the SpO_2?

<90%

TOO LOW
<u>Increase</u> O_2 therapy

Max flow-rate = 2L/min via nasal prongs

Check the SpO_2 after 15 minutes, repeat in an hour.

SpO_2 <90%

Consider increasing respiratory support

Maximise oxygen support and consider escalating respiratory support if available. (e.g. CPAP, high flow)

Monitor SpO_2 continuously.

90-95%

GOOD
<u>Continue</u> O_2 therapy

Continue routine monitoring

>95%

TOO HIGH
<u>Decrease</u> O_2 therapy

Check the SpO_2 after 15 minutes, repeat in an hour.

Repeat as above

Routine SpO_2 monitoring

Monitor SpO_2 and vital signs at least 4 times daily in all patients, and 4-6 hourly while on oxygen

- Aim to keep SpO_2 between 90-95% at all times
- Monitor more frequently in patients with severe respiratory distress (preferably continuously)
- Recheck SpO_2 immediately if patient develops apnoea, worsening respiratory distress, altered conscious state, or other signs of clinical deterioration
- Consider oxygen therapy to maintain SpO_2 ≥95% in special cases (e.g. severe anaemia, shock)

Document SpO_2 and oxygen flow rate on a monitoring chart

Clean the nasal prongs each time you check the SpO_2 to ensure they are patent
