# Supplementary material for: Improving Hospital Oxygen Systems for COVID-19 in Low-Resource Settings: Lessons From the Field
Source: Glob Health Sci Pract. 2020 Dec 23;8(4):858–62. doi: 10.9745/GHSP-D-20-00224 (PMC7784072; doi:10.9745/GHSP-D-20-00224)
Supplement: 20-00224-Graham-Supplement1.pdf [file 20-00224-Graham-Supplement1.pdf]

Supplement to: Graham HR, Bagayana SM, Bakare AA, et al. Improving hospital oxygen systems for COVID-19 in low-resource settings: lessons from the field. Glob Health Sci Pract. 2020;8(4). https://doi.org/10.9745/GHSP-D-20-00224

Clinical use of Oxygen: training package

Version 2.0 (May 2020)

Contents

Notes for Trainers **2**
Evidence-based Teaching 2
How to use this Training Package? 3
Module 0 – Practical task-based teaching techniques **5**
Module Outline 5
Module 1 – Hypoxaemia and Using a Pulse Oximeter **7**
Module Outline 7
Module 2 – Oxygen decision making **9**
Module Outline 9
Scenarios 11
Module 3 – How to use oxygen equipment **14**
Module Outline 14
Module 4 – Pneumonia: Assessment and Classification **16**
Module Outline 16
Scenarios 18
Frequently Asked Questions (FAQs) **19**
Hypoxaemia and pulse oximetry 19
Oxygen therapy 22
Oxygen Equipment – delivery devices 25
Oxygen equipment - Concentrators and other oxygen sources 29
Pneumonia 30
Job Aids (clinical algorithm charts) **33**
Weekly Equipment Checklist **37**
References **39**

Feedback

We welcome feedback about this training package, and your suggestions will help us make the training package better. Please email Dr Hamish Graham - hamish.graham@rch.org.au. For more resources: https://bit.ly/O2clinical.

This training package is based on WHO guidelines and recommendations. More information can be found in the ***WHO Pocketbook of Hospital Care for Children***, and the ***Clinical Use of Oxygen manual***. You can obtain more training resources, and the evidence behind these recommendations, on at http://www.ichrc.org.

Notes for Trainers

This training package is designed as a tool for trainers to help them teach doctors, nurses, and other health care providers about **the use of pulse oximetry and oxygen therapy for children and newborns**. Please **review all the material** thoroughly before conducting training, so that you can adapt the teaching to be most relevant to your learners.

Evidence-based Teaching

The tools in this package are designed using **evidence-based teaching** methods. We recognise that learning is an active process requiring learners build on their existing knowledge and experience[1], and that learning is a social activity whereby learners are strongly influenced by peers, social pressures and the environment[2].

Each activity is framed around David Merrill's "First Principles of Instruction"[3,4]. Merrill's first principles were derived from the most effective instructional design theories with the criteria that they had to promote **more effective, efficient, or engaging learning**. Merrill's five principles involve four action steps (activation, demonstration, application, integration) focussed on a defined task (Fig 2)[3]. The task should be a 'real-world' problem, which begins simply and increases in complexity as learners advance. This is summarised in Table 1.

Table 1 Evidence-based teaching using Merrill's "First Principles"

Principle and Teaching Task	Example Methods
**Activation**<br>*Help learners build on existing knowledge and skills*	• Ask about past experiences & review current knowledge (use peer interaction)<br>• Describe guideline / procedure (focus on task, not detailed background)
**Demonstration**<br>*Guide learners through examples, from simple to more complex*	• Show examples of the concept or procedure (video, in-person, peer demonstration)<br>• Use multiple examples of increasing complexity<br>• Ask for feedback on what is observed (peer discussion)
**Application**<br>*Coach learners using practice and feedback, from simple to complex*	• Practice sessions where learners try out new skill<br>• Ask what was done well, what could be. Link feedback to the skill described (i.e. explicit performance criteria)<br>• improved (peer evaluation, peer feedback)<br>• Small group facilitated case discussions (guided by experts)<br>• Case studies with audience response systems<br>• Practice on patients (with consent)<br>• Simulation
**Integration**<br>*Help learners reflect on how they will apply their new knowledge and skills*	• Self-evaluation<br>• Ask what will you do differently<br>• Commitment to change/intent to practice<br>• Brainstorm what would make it easier to perform in real-life<br>• Create/distribute job aids<br>• Audit to understand actual care

How to use this Training Package?

This training package includes a selection of MODULES and a mix of visual, audio, and written resources.

Each module is designed for a 45-60 minute teaching session. Each module focuses on a specific TASK, and includes suggested activities for ACTIVATION (~10-15 mins), DEMONSTRATION (~15-20 mins), APPLICATION (~15-20 mins), and INTEGRATION (~5-10 mins).

Choose the most relevant activities for your learners, and do them carefully and deliberately. DO NOT try and do every activity that is suggested. This will result in lack of time and superficial learning. You can always repeat that module at a later session using a different selection of activities.

Use the teaching session to focus on the most important learning points. DO NOT try and cover all the theory. This will result in distraction from the most important information.

Use the **Additional Resources** and **Frequently Asked Questions (FAQs)** to help you prepare. These are primarily intended for use by teachers (not learners), but you may like to provide it to learners to refer to after the course.

Tips and Optional extras

- **If learners are shy** about answering questions in large group – ask everyone to discuss the question in pairs, then get them to report back to the group.
- **Look for ways to involve the learners in teaching and evaluating each other**. You will find many activities that seek to involve learners through discussion, demonstration, practice and feedback. These are very effective teaching methods.
- **ACTIVATION** activities can be supplemented by readings. This is best done before the teaching session, so that learners can reflect on this during the session.
- **DEMONSTRATIONS** can be done using pictures, video, simulation dolls/dummies, or real-life patients. Don't worry about doing it perfectly – the important thing is to show it.
- **APPLICATION** activities can be done using scenarios or real-life patients. The important thing is that learners are given a supportive environment to practice and receive helpful feedback (from teachers and peers).
- **INTEGRATION** ideally involves engaging learners in practical steps that will help them (and others) apply the learning – e.g. create a visual prompt and display it on the wall. At the very least, it should involve a chance to reflect on what has been learned and what they will do differently now.
- **Short videos** can be useful if they are the best way to demonstrate something. Keep them brief, and make it clear what you want learners to be observing.
- **Note taking** has been shown to improve student learning (and taking notes with pen and paper is better than taking notes on computer)[5,6]. Providing a brief class outline can be helpful (e.g. one page summary of key headings, or copy of key image/diagram/formula).
- **Handouts** can be a useful for learners to refer back to at a later date, but they can be distracting during a class. Consider making the **Additional Resources** and **FAQs** available for learners after training is finished.
- **PowerPoint** does not increase student learning, even though many students like it[7]. Research shows that PowerPoint is a difficult tool to use well, and most teachers use it badly causing distraction, confusion or oversimplification[7]. PowerPoint seems to be most useful to show pictures or videos that demonstrate a key teaching point. For that reason, this teaching material does not require the use of PowerPoint or computer, and we discourage trainers from using it unless it is really the best way of demonstrating something.

The "Learning Pyramid"

What percentage of information is retained through the following teaching methods? Fill in the boxes below (answers are in Module 0 notes).

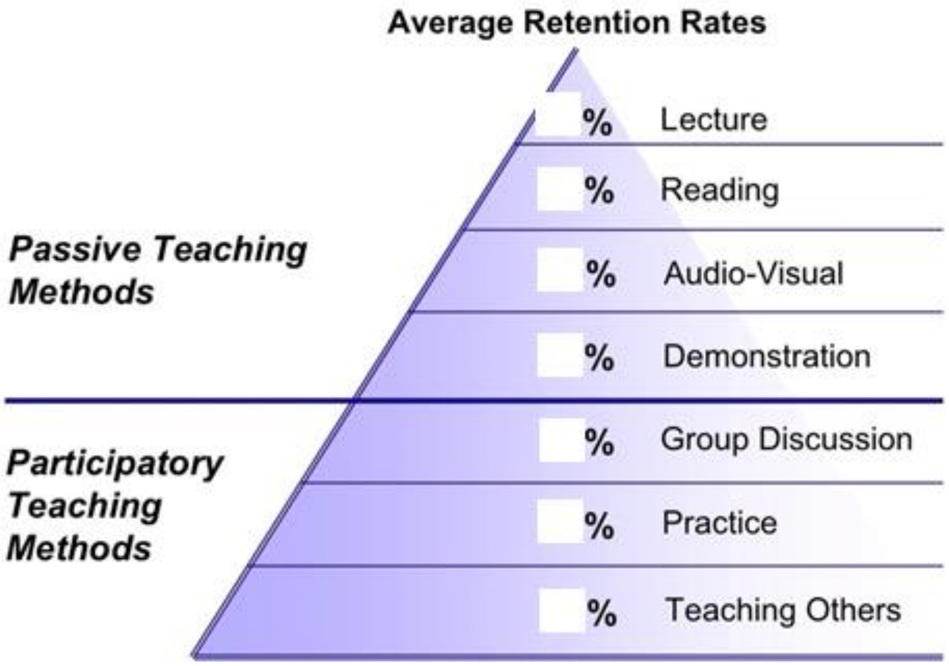

Attributes of a good teacher

Teaching clinical skills can be challenging for doctors and nurses – especially if we have never been taught how to teach. This training package uses evidence-based teaching methods to promote the most effective, efficient and engaging learning. But it also requires trainers that are passionate, caring, and want to help learners reach their potential. Here are some tips, and quotes on what makes a good teacher.

The 5 Essential Attributes of a Good Teacher

Passionate. *Good teachers love what they are teaching.*

Caring. *Good teachers want to help learners learn.*

Challenging. *Good teachers gently push learners to the next level.*

Respectful. *Good teacher recognise learners' humanity and intelligence.*

Practical. *Good teachers make learning relevant to learners.*

Tell me and I forget,
Teach me and I may remember,
Involve me and I learn.
Benjamin Franklin

The mediocre teacher tells.
The good teacher explains.
The superior teacher demonstrates.
The great teacher inspires.
William Arthur Ward

Module 0 – Practical task-based teaching techniques

Teaching tips

- This module is to help trainers understand the principles of task-based teaching. It is an opportunity for facilitators to model the teaching techniques and behaviours that are being taught.

Equipment needed

- Notes for Trainers handout
- Whiteboard (or large paper for notes)
- (Optional: computer with projector)

Module Outline

<table>
<tr><th>Teaching Task</th><th>Activities</th></tr>
<tr><td>

Activation
Help learners build on existing knowledge and skills

~5-10 minutes

</td><td>

INTRODUCE the module.

Task: to be able to use practical task-based teaching techniques.

Objectives:
1. To understand how people learn best
2. To develop practical task-based teaching skills
3. To increase confidence and motivation to use effective teaching skills for future teaching

ASK learners what different methods of teaching they are familiar with
- *I know many of you have experience doing training – what different teaching methods have you used?*
  [List answers on the board]

</td></tr>
<tr><td>

Demonstration
Guide learners through examples, from simple to more complex

~10-15 minutes

</td><td>

SHOW the learning pyramid (p5).

ASK learners to give examples of each type of teaching method.

ASK learners to guess what proportion of information is retained from each of the different teaching methods. (They can fill out the blank pyramid on page 4)

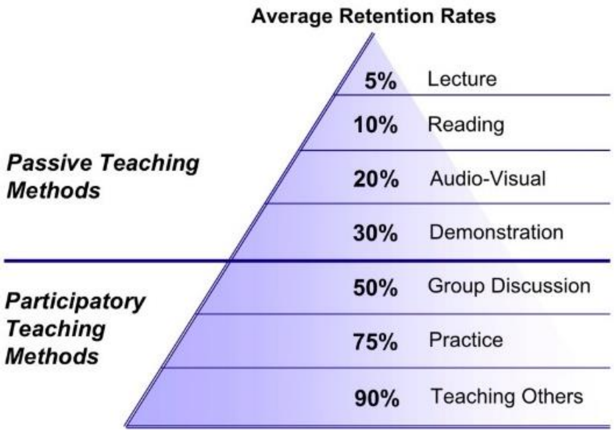

EMPHASISE that participatory teaching methods require learners to do the work, and are generally more effective.

</td></tr>
<tr><td>

Application

</td><td>

PRACTICE TASK:
- *Imagine you are teaching someone who has never worn lace-up shoes how to tie their shoelaces.*

</td></tr>
</table>

Coach learners using practice and feedback, from simple to complex<br><br>~15 minutes	*Try using each of the different teaching methods (lecture, reading, audio-visual, demonstration, group discussion, practice, teaching others) to teach your neighbour how to tie their shoes*.<br><br>[Learners practice in small groups]<br><br>**OBSERVE** each group check that people understand the different teaching methods. Provide **FEEDBACK**, with reference to the Learning Pyramid and the different teaching methods.<br><br>**EMPHASISE**<br>• participatory teaching methods generally get better results than passive teaching methods – but they often require more time and energy.<br>• this doesn't mean that some methods should never be used; they may all be useful to some degree for some purposes. The important thing is to use the right combination of methods to achieve effective, efficient, and engaging instruction for the specific teaching purpose.
**Integration**<br>*Help learners reflect on how they will apply their new knowledge and skills*<br><br>~5-10 minutes	**TEST** key points.<br>• *What is the difference between 'active' and 'passive' teaching methods? Can you give an example?*<br>• *What methods were most effective in teaching someone to tie their shoelaces?*<br><br>**ASK** learners what they have learnt about teaching, and how it will change their practice.<br>• *What will you do differently after today? How could teaching be done more effectively at your hospital?*

Module 1 – Hypoxaemia and Using a Pulse Oximeter

Teaching tips

- This module should be very practical. If possible, take learners into their ward area to help them work out how they would actually apply these skills in real life.
- <u>Read the *FAQs* before teaching</u>. The *FAQs* contain information on all the major learning points, plus extra information for Advanced Learners.
- Remember the practical task-based teaching strategies described in *Notes for Trainers*.

Equipment needed

- Pulse oximeters and probes (ideally 1 oximeter per 6 learners)
- WHO Pocketbook of Hospital Care for Children
- Ward observation charts
- Whiteboard or paper for notes
- Job Aids (clinical algorithm charts)
- Optional: pulse oximetry video (https://www.youtube.com/watch?v=SLkvAA75uTE)

Module Outline

Teaching Task	Activities
**Activation**<br>*Help learners build on existing knowledge and skills*<br><br>~5-10 minutes	**INTRODUCE** the module.<br>(Task): *By the end of this module you will be able to* confidently use a pulse oximeter.<br><br>(Objectives): *This module will help us…*<br>1. understand the importance of pulse oximetry in detecting hypoxaemia<br>2. develop our skills using pulse oximeters and solving common problems<br>3. increase our motivation to use pulse oximeters in clinical practice<br><br>**ASK** learners what they want to get out of the session<br>• *I know many of you have experience with pulse oximeters – what do you want to learn today?*
**Demonstration**<br>*Guide learners through examples, from simple to more complex*<br><br>~10-15 minutes	**ASK** learners about hypoxaemia (*optional: discuss in pairs*)<br>• *What is hypoxaemia*?<br>• *How do we know if a child has low blood oxygen*? [write on paper]<br>• *Who has used pulse oximeters? Who do you use it on?*<br><br>**DISCUSS** the signs of hypoxaemia [see *FAQs* and *Pocketbook* p76-77]<br><br>**EMPHASISE:** *Even the best clinician will miss one third (1/3) of children with hypoxaemia if they rely just on clinical signs – pulse oximetry is essential!"*<br><br>**EMPHASISE**: *All sick children should have pulse oximetry performed on admission – especially those with signs of respiratory or severe illness.*<br>- - - - -<br>**SHOW** learners a pulse oximeter.<br>• *Pulse oximeter... Screen. On/off button. Battery. Blue connector.*<br>• *Oximeter probe… Light & sensor. Blue connector & wire (fragile).*<br>• *Insert the battery (if not already in place).*<br>• *Connect the probe to the oximeter. Be careful with the pins. Do not disconnect unnecessarily or you may damage the pins.*<br>• *Turn the oximeter on. Now you are ready to use it on a patient.*

Application
Coach learners using practice and feedback, from simple to complex

~20-25 minutes

PRACTICE in small groups: Learners should take turns doing pulse oximetry on each other.

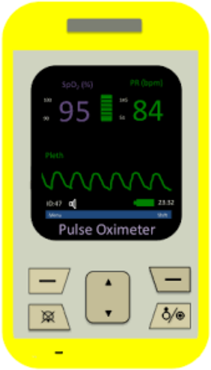

ASK: *what do you see on the screen?*
Explain each of the things displayed?

- Saturation (SpO_2)
- Pulse rate (PR)
- Pulse indicator
- Pleth (waveform)
- Battery

- - - - -

SHOW learners how to get a good reading and troubleshoot problems using the "5 steps". [Optional: show pulse oximetry video]

- *Sometimes it can be difficult to get a good reading on a child. Children may be moving or upset, or their hands can be cold.*

T
Talk through the 5 steps, explain *how* to do it and *why* it is important:

(1) prepare the oximeter and patient;
(2) place the probe gently on the finger;
(3) wait until there is a good pulse waveform;
(4) document SpO2 and oxygen flow rate on a monitoring chart;
(5) remove the probe and wipe it clean.

PRACTICE in small groups: Learners should take turns practicing the 5-step method (including documentation), and giving each other **FEEDBACK**.

Integration
Help learners reflect on how they will apply their new knowledge and skills

~5-10 minutes

TEST learners on the key points (see FAQs for answers).

- *What is hypoxaemia?*
- *Which patients should we do pulse oximetry on?*
- *What information does pulse oximetry tell us?*
- *What is the importance of the pulse waveform (pleth)?*
- *What can we do if we are having difficulty getting a good reading?*
- *…*

ASK learners how they will apply what they have learnt today.

- *What will you do differently after today? Or what will you tell your colleagues to do differently?*
- *Where will the oximeter be kept? Who will be responsible for testing every child? How will you ensure that results are documented? How can you help other people remember to do oximetry?*

(Optional: take small groups onto the ward to work out where the oximeter should be kept, and how results will be documented)

Module 2 – Oxygen decision making

Teaching tips

- This module should be very practical. If possible, take learners into their ward area to help them work out how they would actually apply these skills in real life.
- Read the *FAQs* before teaching. The *FAQs* contain information on all the major learning points, plus extra information for Advanced Learners.
- This module assumes basic knowledge about hypoxaemia (*Module 1 – hypoxaemia and using a pulse oximeter*).
- Remember the practical task-based teaching strategies described in *Notes for Trainers*.

Equipment needed

- How to use Oximetry & Oxygen Charts
- Scenarios [optional ipad/iphone with SimMon]
- WHO Pocketbook of Hospital Care for Children
- Ward observation charts
- Whiteboard (or paper) for notes
- Job Aids (clinical algorithm charts)

Module Outline

Teaching Task	Activities
**Activation**<br>*Help learners build on existing knowledge and skills*<br><br>~5-10 minutes	**INTRODUCE** the module.<br>(Task): *By the end of this module you will be able to* make good decisions about starting, monitoring, and stopping oxygen therapy.<br><br>(Objectives): *This module will help us…*<br>1. understand the importance of hypoxaemia and oxygen therapy<br>2. develop skills in starting, monitoring, and stopping oxygen therapy<br>3. increase motivation to monitor for hypoxaemia in practice<br><br>**ASK** learners what they want to get out of the session<br>• *I know many of you have experience with using oxygen – what do you want to learn today?*
**Demonstration**<br>*Guide learners through examples, from simple to more complex*<br><br>~10 minutes	**ASK** learners about their experience using O2.<br>• *What is oxygen therapy*?<br>• *Who should we give oxygen therapy to*? [write on paper]<br><br>**DISCUSS** why oxygen is important for life, and who to give oxygen to [see *Job Aids*, *FAQs* and *Pocketbook* p312-315]<br>- - - - -<br>**SHOW** the *Oxygen Job Aids (clinical algorithm charts)*.<br>Ask people to read them and ask questions.<br>• Using an Oximeter.<br>• Monitoring and Stopping Oxygen Therapy<br>• Monitoring and Stopping Oxygen Therapy (Preterm / Small)
**Applicati**[illegible]<br>*Coach learners using practice and*	[illegible]**ICE** in small groups, using SCENARIOS (next page).

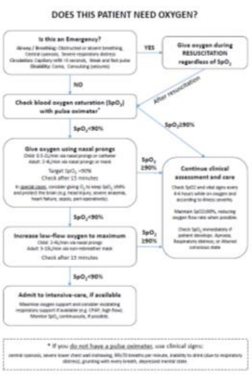

feedback, from simple to complex<br><br>~20-25 minutes	*We are now going to do some scenarios. I will give you a scenario and you need to work out the answers using your Oxygen Job Aids, Pocketbook, and by discussing it with your colleagues.*<br><br>[See SCENARIOS below]<br><br>**OBSERVE** each group and provide **FEEDBACK** based on the *Oxygen Job Aids* and *FAQs*.<br><br>**EMPHASISE** the importance of documentation.
**Integration**<br>*Help learners reflect on how they will apply their new knowledge and skills*<br><br>~5-10 minutes	**TEST** learners on the key points (see FAQs for answers).<br>• *Who should we give oxygen therapy to?*<br>• *What about Special Cases? Emergencies?*<br>• *What SpO2 do we aim for in children and adults?*<br>• *What SpO2 do we aim for in preterm/small neonates? Why?*<br>• *How much oxygen do I give?*<br>• *How do I know when to decrease or stop giving oxygen therapy?*<br>• *What do I do if the child or parents are scared of the oxygen?*<br>• *…*<br><br>**ASK** learners how they will apply what they have learnt today.<br>• *What will you do differently after today? Or what will you tell your colleagues to do differently?*<br>• *How will you make sure the SpO2 and oxygen flowrates is recorded?*<br>• *What will help other people give oxygen appropriately? Do you need posters or some other reminders on the ward? Are there other things that would make it easier on your ward?*<br><br>*(Optional: take small groups onto the ward to review patient records, and practice recording oxygen and flowrate)*

Scenarios

Read from the 'Facilitator' column – let learners work out the correct answers by looking at the handouts, charts and discussing it with each other.

FACILITATOR QUESTIONS	LEARNER ANSWERS
**SCENARIO 1: (basic)**<br><br>i. 3yo boy (Joseph) presents with difficult breathing. What would you do to work out whether he needs oxygen?<br>[If asked: HR 150, RR 60, SpO2 94%, PCV/EVF 30%, Hb 10g/dl, Malaria test negative, mild lower chest wall indrawing, feeding okay]<br><br>ii. What if her SpO2 was 88%?<br>iii. What if she had seizures?<br><br>iv. What if her PCV was 13%?<br>v. What if her Malaria test was positive?	<u>[Let learners work these answers out by themselves]</u><br><br>i. Check: ABC, HR, RR, SpO2, conscious state.<br>Consider: anaemia, malaria, feeding, oxygen<br><br>=> No, she does not need oxygen currently.<br><br>[diagnose pneumonia, give antibiotic and advice]<br><br>ii. Give oxygen if SpO2<90%.<br>iii. Seizures are an Emergency sign, give oxygen during resuscitation.<br>iv. Severe anaemia is a Special Case, aim SpO2≥94%<br>v. Malaria – don't give oxygen (unless SpO2<90%, emergency signs, special case).
**SCENARIO 2: (child)**<br><br>i. 2yo girl (Aysha) admitted to ward with a fever and fast breathing. What will you do?<br>[If asked: HR 150, RR 60, SpO2 88%, PCV 30%, Malaria test positive, severe lower chest wall indrawing, poor oral intake]<br><br>ii. Aysha's mother doesn't want oxygen – she says it kills children. What do you do now?<br><br>iii. You have started oxygen and after 15 minutes the SpO2 is 98%. What do you do now?<br><br>iv. After 2 hours Aysha's mother says she is worried that Aysha is not waking up. What will you do?<br>[If asked: severe subcostal indrawing, drowsy, HR 170, RR 70, SpO2 80%, prongs are off]<br><br>v. You are finishing your shift. What will you handover?<br>[NB: emphasise documentation]<br><br>vi. You return after 2 days. Aysha is doing well. How do you know if she can stop oxygen therapy?<br>[If asked: HR 120, RR 40, SpO2 99% 0.25L O2]	<u>[Let learners work these answers out by themselves]</u><br><br>i. Check: ABC, HR, RR, SpO2, conscious state.<br>Consider: anaemia, malaria, feeding, oxygen<br>=> Start O2 therapy, 1L/min via nasal prongs.<br>[diagnose severe pneumonia & malaria, give antibiotic, anti-malarial, consider NG fluids]<br><br>ii. Reassure, explain, and demonstrate using pulse oximetry.<br><br>iii. Continue oxygen, check SpO2 after 1 hour<br>[NB: you may decrease the oxygen flowrate but do not stop oxygen suddenly]<br><br>iv. Airway, breathing, circulation (ABC)<br>Check prongs [dislodged], clean and readjust and redo taping [SpO2 increases to 92%].<br>Consider changing flow rate [not necessary].<br>Consider medical review.<br><br>v. E.g. "Asha has severe pneumonia & malaria, requiring oxygen (1L/min), antibiotics, antimalarial." Document SpO2 and O2 flowrate.<br><br>vi. Check SpO2, oxygen (etc.).<br>Reduce (or stop) oxygen if SpO2≥95%.<br>Recheck after 1 hour.

vii. When can Aysha be discharged?	vii. Consider discharge if the SpO2 is stable ≥90% without oxygen for 24 hours.
**SCENARIO 3: (neonatal)**<br><br>i. Newborn baby with fast and difficult breathing at 1 hour. What do you want to do?<br>[If asked: 1800g, HR190, RR80, grunting, severe subcostal indrawing, SpO2 85%]<br><br>ii. You start oxygen at 0.5L/min, and the SpO2 is now 98%. What do you do now?<br><br>iii. After 1 hour, the SpO2 is 94%, RR70. What will you do now?<br><br>iv. After 2 hours baby has increased grunting and 5 second pauses in breathing. What next?<br>[If asked: RR90, severe subcostal indrawing, SpO2 78%, signs of pneumothorax]<br><br>v. You increased the O2 to 2L/min (max for infant) and drained the pneumothorax. Now: RR70, improved subcostal indrawing, SpO2 98%. What do you do next?	[Let learners work these answers out by themselves]<br><br>i. Check: HR, RR, SpO2, Temp, birth weight<br>Consider: risk for sepsis, preterm care (start early breast feeding, keep warm, kangaroo mother care).<br>Start O2 therapy, 0.5-1L/min via nasal prong<br><br>ii. Decrease O2 to 0.25L/min [aim SpO2 90-95%]. Check again in 1 hour<br><br>iii. Continue O2 at same rate [aim SpO2 90-95%]<br><br>iv. Check SpO2 [78%]<br>Increase oxygen [max 2L/min]<br>Check for pneumothorax, and drain.<br><br>v. Decrease O2 to 0.25-0.5L/min, check again<br>[aim SpO2 90-95%]

SCENARIO 4: (documentation)

Copy the information below onto local observation charts (*Alternative: write on whiteboard*).

Show the chart and **Ask** "what is missing?" "what could be done better?"

Facilitator input

- Chart 1 - Missing SpO2 but is currently on oxygen

Date Time	1/3 1800	1/3 2400	2/3 0900	2/3 1200	2/3 1800	3/3 0900	4/3 1800	4/3 1800
**RR**	60	60	70	60	60	50	70	60
**SpO2**								
**O2**	yes			no				yes

- Chart 2 - Missing oxygen flow rates

Date Time	1/3 1800	1/3 1800	2/3 0900	2/3 1200	2/3 1200	2/3 1800	2/3 1800	3/3 0900
**RR**	60	60	70	60	80	90	70	60
**SpO2**	88%	98%	88%	90%	96%	96%	94%	92%
**O2**								

- Chart 3 - Missing oxygen flow rate changes

Date Time	1/3 1800	1/3 2400	2/3 0400	2/3 0800	2/3 1200	2/3 1600	2/3 1800	2/3 2400
**RR**	60	60	70	60	80	90	70	60
**SpO2**	88%	98%	88%	90%	96%	96%	94%	92%
**O2**	-	1L	0.5L	1.5L	2L	1L	-	-

- Chart 4 - Documentation without action

Date Time	1/3 1800	1/3 1800	1/3 2400	2/3 0400	2/3 0800	2/3 1600	2/3 1800	2/3 2400
**RR**	60	60	70	90	90	70	40	0
**SpO2**	88%	98%	88%	84%	86%	80%	62%	0%
**O2**	-	0.5L	0.5L	0.5L	0.5L	0.5L	0.5L	0.5L

Correct learner actions

- Chart 1
  No SpO2 documented, no flow rates documented.
  Unable to know why they started on oxygen, were stopped, and restarted.
  Should regularly document SpO2 and flow rate in litres per minute.

- Chart 2
  SpO2 documented, but no flow rates.
  Unable to know whether the child was on oxygen or how much.
  We don't know if the child is getting sicker or better.
  Should include the flow rate.

- Chart 3
  SpO2 and flow rates documented every 3-4 hours. But no changes in flow rate.
  Unable to know why the flow rate was changed.
  Document every SpO2 tested, and make change in flow rate clear.

- Chart 4
  SpO2 and flow rate documented every 4 hours, including when it was started.
  Good documentation – it shows a child getting sicker and sicker and dying.
  But no action.

Module 3 – How to use oxygen equipment

Teaching tips

- This is a large module – and is best done over two sessions (or one longer session). The teaching in this module should be directly connected to what learners will be doing in their daily practice. Consider taking learners into their usual work environment to practice.
- Read the *FAQs* before teaching. The *FAQs* contain information on all the major learning points, plus extra information for Advanced Learners.
- This module assumes basic knowledge about hypoxaemia (*Module 1 – hypoxaemia and using a pulse oximeter; Module 2 – oxygen decision making*).
- Remember the practical task-based teaching strategies described in *Notes for Trainers*.

Equipment needed

- Oxygen concentrator (or cylinder)
- Nasal prongs and nasogastric tubes (size 6 or 8 French)
- Sureflow flowmeter device
- Glass/plastic beaker
- WHO Pocketbook of Hospital Care for Children
- Weekly Equipment checklist

Module Outline

Teaching Task	Activities
**Activation**<br>*Help learners build on existing knowledge and skills*<br><br>~5-10 minutes	**INTRODUCE** the module (including the task and objectives).<br>(Task): *By the end of this module you will be able to* confidently use an oxygen concentrator, flowmeter assembly, and oxygen delivery devices.<br><br>(Objectives): *This module will help us…*<br>1. understand oxygen equipment (nasal prongs, flowmeters & concentrators)<br>2. develop skills using nasal prongs, flowmeters & concentrators<br>3. increase our motivation to use and look after equipment appropriately<br><br>**ASK** learners what they want to get out of the session<br>• *I know many of you have experience using oxygen equipment – what do you want to learn today?*
**Demonstration**<br>*Guide learners through examples, from simple to more complex*<br><br>~10-15 minutes<br><br>- - -<br>~15-20 minutes	**ASK** learners about their experience using oxygen, and what kind of delivery devices they have used (*optional: discuss in pairs*)<br><br>• *What can you use to give oxygen to a child*?<br>[E.g. mask, head box, nasal prongs, nasal catheter, nasopharyngeal catheter]<br>• *Which are best*?<br>[write on paper – discuss pros and cons]<br><br>**EMPHASISE:** *Nasal prongs are easy to use, effective, and comfortable.*<br><br>Ask a volunteer to **SHOW** how to use nasal prongs (and catheters). [p11, p313]<br>[Optional: **SHOW** how to clean and disinfect nasal prongs for re-use]<br><br>- - - - -<br>**SHOW** learners the *Equipment Checklist*, and ask them to identify the equipment.

- Sureflow® flowmeter assembly: 5 x 2LPM flowmeters; Inlet (connector on both sides); Outlets.
- Oxygen Concentrator: On/off button; Flowmeter; Outlet; Low oxygen alarm; Low power alarm; Hour meter; External dust filter; Fire safety.
- Bubble test: Demonstrate, *", we need to check regularly to make sure it is working safely and effectively"*

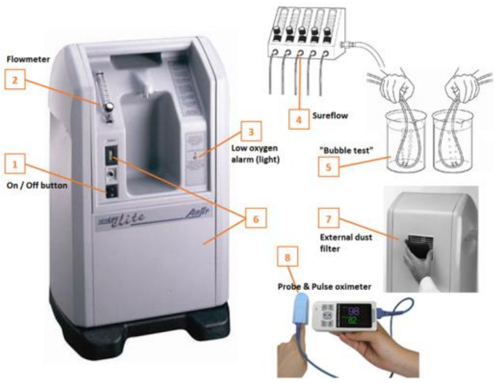

ASK: *How does an oxygen concentrator work?* [See *FAQs* for answers]

Application
Coach learners using practice and feedback, from simple to complex

~35-45 minutes

PRACTICE in small groups. Learners should take turns practicing and giving feedback:
- using nasal prongs and nasal catheters
- doing the 'Bubble test'
- titrating oxygen on a Sureflow device
- doing the Equipment Checklist.

OBSERVE each group and provide **FEEDBACK**. Make sure that every person gets a chance to use all the equipment – and do the complete Equipment Checklist.

(*Optional: take small groups onto the ward to practice using nasal prongs and titrating oxygen using Sureflow® device on patients with consent)*

Integration
Help learners reflect on how they will apply their new knowledge and skills

~10 minutes

TEST learners on the key points (see FAQs for answers).
- What is the preferred oxygen delivery device?
- How do you know that the patient is receiving oxygen correctly?
- What does the loud alarm on the concentrator mean? What does the light on the concentrator mean?
- Why is there a "no smoking" sign on the concentrator?
- What do users need to do to look after the concentrator? What will you do if you have a problem?

ASK learners how they will apply what they have learnt today.
- *What will you do differently after today? Or what will you tell your colleagues to do differently?*
- *Who will be responsible for cleaning nasal prongs? Doing the Equipment Checklist?*
- *How will new staff be trained to use nasal prongs and other oxygen equipment? What will help other people remember to do oximetry? Do you need posters or some other reminders on the ward? Are there other things that would make it easier to do oximetry on your ward?*

(Optional: take small groups onto the ward to work out where the nasal prongs will be stored, cleaned and dried)

Module 4 – Pneumonia: Assessment and Classification

Teaching tips

> **Equipment needed**
> - WHO Pocketbook of Hospital Care for Children (chapter 4)
> - Whiteboard or large paper for notes
> - Videos and/or images demonstrating respiratory signs

- This module contains introductory and advanced content about the assessment and classification of pneumonia. Advanced learners may be able to move quickly to the SCENARIOS. Less advanced learners should master the basic signs and symptoms of pneumonia first, and do the SCENARIOS in another session.
- The teaching in this module should be directly connected to what learners will be doing in their daily practice. Consider taking learners into their usual work environment to practice.
- This module links with content in Module 1 (hypoxaemia) and Module 2 (oxygen). *See also the Notes for Trainers on page 2 and FAQs in the Appendix.*

Module Outline

Teaching Task	Activities
**Activation**<br>*Help learners build on existing knowledge and skills*<br><br>~5-10 minutes	**INTRODUCE** the module (including the task and objectives).<br>(Task): *By the end of this module you will be able to* confidently assess and correctly classify pneumonia.<br><br>(Objectives): *This module will help us…*<br>• To understand the symptoms and signs of pneumonia<br>• To develop skills in assessing children with breathing problems<br>• To increase motivation to classify pneumonia and severe pneumonia<br><br>**ASK** learners what they want to get out of the session<br>• *I know many of you have experience with pneumonia – what do you want to learn today?*
**Demonstration**<br>*Guide learners through examples, from simple to more complex*<br><br>~10-15 minutes	**ASK** learners about the symptoms and signs of pneumonia [p76-77]. (*optional: discuss in pairs*) [write on paper]<br>• *What symptoms may children with pneumonia have?*<br>• *What clinical signs would you look for? How do you assess for this?*<br>(*Advanced: compare pneumonia signs/symptoms to other respiratory illnesses – p77-78*)<br><br>**SHOW** each of the signs using video (or physical demonstration, or images) (see FAQs). *“Now we are going to see examples of each of these signs. Please ask if anything is not clear.”*<br>• Difficult breathing; Fast breathing (age <2 months ≥60/min; age 2-12 months ≥50/min; age 1-5 years ≥40/min); Chest indrawing; Hypoxaemia (SpO2 <90%) or Central Cyanosis; Grunting; Danger signs (Lethargy/reduced conscious state, Convulsions, Inability to feed)<br><br>**EMPHASISE**: *‘danger signs’ are an emergency. Seek extra help and give emergency treatment before completing your assessment. Ch1.*]<br><br>**ASK** learners about classifying the severity of pneumonia [p81].

	• *How can I classify pneumonia by severity?* (refer to the updated WHO classification – pneumonia vs severe pneumonia) [Write on paper].<br><br>**EMPHASISE** the importance of recognising and documenting the severity of pneumonia so that appropriate treatment can be given.
**Application**<br>*Coach learners using practice and feedback, from simple to complex*<br><br>~20-25 minutes	**PRACTICE** in small groups, using video clips and SCENARIOS (next page).<br><br>Groups should discuss, decide, and document. Then report to group.<br><br>*(Alternative: use images, or real-life patients on the ward with consent)*<br><br>**OBSERVE** each group and provide **FEEDBACK** based on the *WHO Pocketbook.*
**Integration**<br>*Help learners reflect on how they will apply their new knowledge and skills*<br><br>~5-10 minutes	**TEST** learners on the key points (see FAQs for answers).<br>• How do you distinguish between pneumonia and severe pneumonia?<br><br>**ASK** learners how they will apply what they have learnt today.<br>• *What will you do differently after today? Or what will you tell your colleagues to do differently?*<br>• *What will help other people assess children with cough and difficult breathing appropriately? Are there other things that would make it easier on your ward?*<br><br>*(Optional: take groups onto the ward to review patients with consent)*

Scenarios

FACILITATOR QUESTIONS	LEARNER ANSWERS
1. Addy is an 18 month old boy with one week of fever and cough. He is unable to drink any fluids. This is what he looks like on examination. What is your diagnosis?<br><br>**SHOW VIDEO Case 1** from pneumonia drills<br><br>[If asked: SpO2 88% RA, PR 160, temperature 37.5 degrees (39 degrees one hour ago), decreased breath sounds on the left side]	Answer: Severe pneumonia<br>**ASK** why: inability to drink, video shows tachypnoea about 60 breaths/min and severe chest indrawing
2. Bebi is a 2 year old with cough, fever and runny nose for 3 days. He is able to eat and drink normally. This is what he looks like on examination. What is your diagnosis?<br><br>**SHOW VIDEO Case 2** from pneumonia drills<br><br>[SpO2 98% RA, temperature 37 degrees, HR 100, chest clear]	Answer: Cough/common cold<br>**ASK** why: no tachypnoea or chest indrawing
3. Caty is a 2 year old girl with cough, fever and runny nose for 3 days. Her respiratory rate is 45 breaths per minute. What is your diagnosis?<br><br>**SHOW VIDEO Case 3** from pneumonia drills<br><br>[SpO2 95%, temperature 37.5 degrees, HR 130, widespread crackles on auscultation of the chest]	Answer: pneumonia<br>**ASK** why: tachypnoea and chest indrawing. No signs of severe pneumonia – still able to drink, saturations >90%
4. Deedee is a 11 month old girl with fever, runny nose and cough for 4 days. Her parents have noticed she is making this noise when she breathes (**PLAY Case 4 recording**) and her skin looks like this (**SHOW Case 4 photo**). What is your diagnosis?<br><br>[SpO2 88% RA, HR 165, RR 60, temperature 37.6 degrees]	Answer: Severe pneumonia<br>**ASK** why: grunting, cyanosis
5. Effi is a 9 month old boy with fever, cough and runny nose. His parents have brought him to see you because his chest looks "different" today. You count his respiratory rate to be 55. What is your diagnosis?<br><br>**SHOW Video Case 5 from** pneumonia drills<br><br>[SpO2 94%, HR 120, Temperature 37.8 degrees, drinking normal amount of fluids and not dehydrated]	Answer: Pneumonia<br>**ASK** why: chest indrawing and tachypnoea
6. Faddy is a 9 month old boy with fever, cough and runny nose for 3 days. He is still able to eat and drink normally. This is what he looks like. What is your diagnosis?<br><br>Show **Video Case 6** from pneumonia drills<br><br>[SpO2 95%, HR 130, RR 35, Temperature 37.9 degrees, not dehydrated]	Answer: cough/common cold<br>**ASK** why: no chest indrawing or tachypnoea, able to eat and drink normally

Frequently Asked Questions (FAQs)

Hypoxaemia and pulse oximetry

What is hypoxaemia?

Hypoxaemia refers to low blood oxygen levels. Pulse oximetry is the standard available method for detecting hypoxaemia. The normal range for arterial oxygen pulsed saturation (SpO2) at sea level is 97-100%, and this is lower in mountainous regions due to the lower partial pressure of oxygen in arterial blood at higher altitudes[8].

Is hypoxaemia really dangerous? Oxygen is essential for human life (see Oxygen FAQs). Hypoxaemia results in the death of cells in our body and lead to organ failure and death. Evidence suggests that hypoxaemia is the biggest risk factor for death in childhood pneumonia.

How common is hypoxaemia? Hypoxaemia occurs in approximately 13% of children presenting to hospitals with severe pneumonia[9]. Higher prevalence of hypoxaemia is noted in high altitude settings, particularly in Asia and Latin America. However, there is huge variation between different levels of facilities and different regions.

What conditions can cause hypoxaemia? Hypoxaemia can be caused by respiratory infections (pneumonia, bronchiolitis) as well as many other conditions, such as sepsis, severe malaria, status epilepticus, trauma, obstetric and neonatal conditions (respiratory distress, apnoea, asphyxia, sepsis). It is also relevant in therapeutic processes such as surgical care and anaesthesia.

What is the best test of hypoxaemia? Pulse oximetry is the best way to detect hypoxaemia in practice. Using clinical signs alone is unreliable as even the best combinations of clinical signs have unacceptably low sensitivity and specificity[10,11]. Use of pulse oximetry can correctly identify 20-30% more children with hypoxaemia than relying solely on clinical signs – preventing both underuse and unnecessary use of oxygen[8].

What are the clinical signs of hypoxaemia? If you don't have pulse oximetry, look for the following signs: very high respiratory rate (>70bpm); severe lower chest wall indrawing; head nodding (infants); grunting with every breath (infants); central cyanosis; decreased conscious state; unable to feed due to difficult breathing (infants).

Signs such as neonatal feeding issues and elevated respiratory rate, are sensitive signs of hypoxaemia but are commonly associated with conditions not involving hypoxaemia and can result in unnecessary oxygen use. In addition, you may miss the sickest children who are so tired that their breathing rate is normal or even low (bradypnoea).

Signs such as cyanosis, head nodding and grunting strongly suggest hypoxia, but relying on these signs will miss many other children who are also hypoxic and may benefit from oxygen.

What is central cyanosis? Central cyanosis is a blue colour to the tongue and lips – and it indicates severe hypoxaemia. Usually SpO2 needs to be below 70% before it is possible to detect cyanosis. In patients with anaemia it is even more difficult to see cyanosis because of lower concentration of red blood cells in the blood.

Peripheral cyanosis is a blue colour to the fingers or toes. It is usually caused by cold weather or peripheral vasoconstriction, and may not indicate hypoxaemia.

What is a pulse oximeter? A pulse oximeter is a device that tells users what the level of oxygen is in the blood (specifically, the oxygen saturation of red blood cells).

What does pulse oximetry tell me? Different pulse oximeter may look different – some are large and sit on the desktop, some attach to complex monitoring machines, some are small and can be held in the hand, and some are so small they clip to a finger. All oximeters will give you similar information, but some may be better quality than others.

The Lifebox® pulse oximeter is a high quality hand-held oximeter that gives the following display:

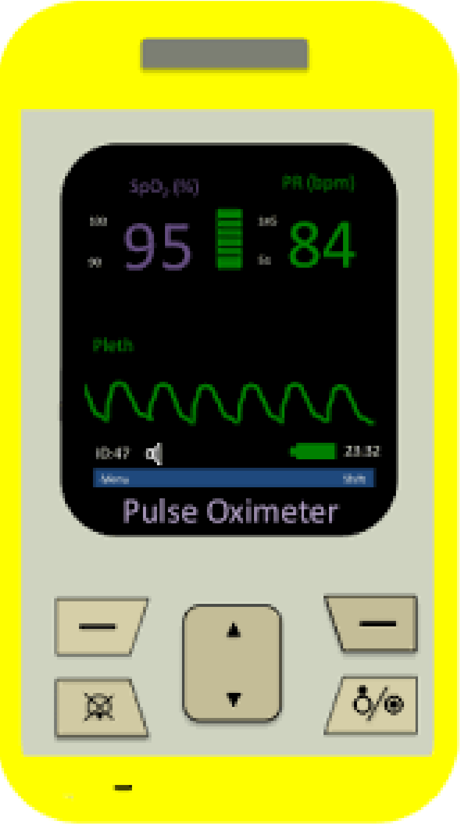

- SpO2 (%) – this tells you the percentage of oxygen in the blood (oxygen saturation of red blood cells).
- PR (bpm) – this tells you the pulse rate (heart rate). There is also a flashing bar to indicate the individual beats. This is important because it tells you whether the oximeter is picking up a good trace. If the beats are not strong and regular, the reading may not be accurate.
- Pleth – this shows you a pulse indicator waveform. This is important because it tells you whether the oximeter is picking up a good trace. If the trace is poor, the reading may not be accurate.
- Battery – this shows you the charge of the battery. If the battery charge is low, the reading may not be accurate.

The display can be changed by pressing the top-right button, and by tipping the oximeter to the side. The other buttons let you change the settings (in the Menu), silence the alarm, and turn on/off.

Pulse oximeters <u>DO NOT</u> tell you the respiratory rate or the haemoglobin level (PCV/Hct/Hb).

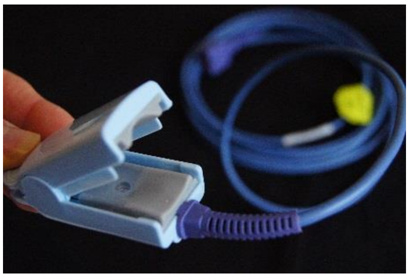

How does a pulse oximeter work? Pulse oximetry measures the percentage of oxygenated haemoglobin in arterial blood. Inside the oximeter probe is a light, which produces red and infrared light when it is placed on the patient. Opposite the light is a detector. Oxygenated and deoxygenated blood allow different amounts of light through. This means that the oximeter can calculate the blood oxygen level based on the amount of light transmitted[8].

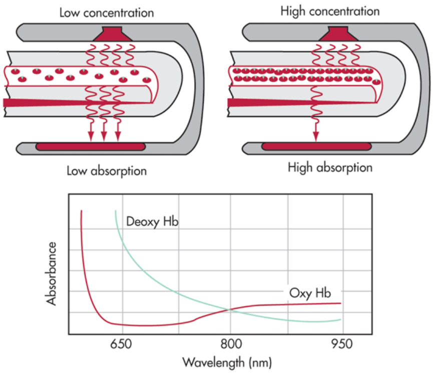

The pulse oximeter will tell you the blood oxygen level, technically called the peripheral capillary oxygen saturation. This is also called the oxygen saturation, SpO2, or “sats” for short. The pulse oximeter will also tell you the heart rate, and display a pulse indicator or waveform. It is important to check for a regular pulse indicator or waveform to make sure you get an accurate reading. The oximeter will give an audible ‘beep’ to indicate each heartbeat, and the ‘beep’ will change tone according to the lower or higher saturations.

How accurate is pulse oximetry? While there is some variation between different oximeters, most good quality oximeters are rated as accurate to +/- 1% at SpO2 levels of 70-100% (compared to blood gas analysis).

What can make it difficult to get a good reading using pulse oximetry?

- The probe may not fit properly because it is the wrong size or location.
  TIP: Try a different sized probe or move the probe to a different finger or toe. Make sure the hands and feet are warm. If they are cold the blood flow will be limited causing the pulse signal to be weak
- If the child is moving, shivering or upset it can be difficult to position the probe correctly.
  TIP: Get the parent to help you by comforting or distracting their child while you measure the oxygen level. Explain what you are doing so that the parent and child are not fearful. Let the child move freely and do not hold the probe tightly as this will stop the blood flow and reduce the pulse signal.
- If the child is very unwell the circulation can be impaired.
  TIP: If there are signs of shock or very poor perfusion, this is an emergency. Resuscitate immediately and don't let pulse oximetry delay treatment.
- Sometimes the problem can be with the oximeter or the probe.
  TIP: Check the oximeter is properly charged. Low charge can cause faulty readings. Check the probe is properly connected. Be careful with the connecting pins. Check the oximeter on your own finger to see if it is functional.

What can make the reading on pulse oximetry inaccurate?

- Nail polish/paint can make it difficult for the oximeter to accurately detect the light. It will usually cause the SpO2 reading to be lower than it really is.
- Strong overhead lights (e.g. theatre lights) shining on an oximeter can result in a higher SpO2 reading.
- Anaemia, including sickle cell disease, does NOT cause inaccurate pulse oximetry readings. However, anaemic children with hypoxaemia will be sicker than non-anaemic children because they have less red blood cells to transport oxygen to tissues.
- Dark skin does NOT cause inaccurate pulse oximetry reading. [Some studies have suggested that oximetry may result in a slightly higher reading (2-3%) in very dark skin when the SpO2 is very low (<70%). However, this is not clinically significant.]

When should I perform pulse oximetry?

Every child admitted to hospital should have SpO2 tested and documented at the time of admission. Ideally, pulse oximetry should be done routinely along with the other routine nursing observations (temperature, heart rate, respiratory rate) throughout the admission.

Additional monitoring is required for:

- Children who develop apnoea, worsening respiratory distress, altered conscious state, or other signs of clinical deterioration – check SpO2 promptly.
- Children who are on oxygen - routinely check SpO2 at least daily (preferably 3-4 hourly).
- Children who are on oxygen with severe respiratory distress – check SpO2 at least 3-4 hourly.
- Children who have had their oxygen flow rate changed in any way (started, stopped, increased, decreased) – recheck one hour after changing.

Oxygen therapy

What is the physiological importance of oxygen?

Oxygen is critical to sustain human life (and other animals). Oxygen is used by cells to release energy from the food that we eat. The cellular respiration formula says that we use carbohydrates ($C_6H_{12}O_6$) together with oxygen (O_2) to produce energy in the form of adenine triphosphate (ATP), carbon dioxide ($6CO_2$) and water (H_2O).

$$C_6H_{12}O_6 + 6O_2 \rightarrow 6CO_2 + 6H_2O + ATP$$

The primary role of our respiratory system is to extract oxygen from the air into our blood, and to expel carbon dioxide from our blood into the air. If we are unable to get enough oxygen to our cells they will die. This is especially important in our 'critical organs' – especially our brain.

What kind of oxygen is used for oxygen therapy?

Air contains 21% oxygen, 78% nitrogen, and 1% other gases (argon, carbon dioxide etc.). In a healthy person, our lungs are easily able to extract enough oxygen from the air for all the cells in our body. In fact, most people would be absolutely fine using just one lung. Medical oxygen contains at least 85% oxygen – more than four times the concentration of air. Medical oxygen can come from oxygen cylinders, oxygen concentrators, or oxygen plants – in doesn't really matter.

It also doesn't really matter whether the concentration of oxygen is 85% or 95% or 100%. It is still much more than the concentration of oxygen in air. And delivery devices, such as nasal prongs, mix the medical oxygen with air so that the gas actually reaching the lungs is more like 30-60% oxygen – which is still significantly more than air (see more about oxygen delivery devices below).

Why is oxygen called an 'essential medication'?

Oxygen is an under-appreciated essential medical therapy that has been saving lives for over 100 years[12]. It is relevant not only for pneumonia and other primary lung diseases, but also many other conditions that result in hypoxaemia (see above), as well as therapeutic processes such as surgical care and anaesthesia. Given its wide and successful use for over a century, it is remarkable that oxygen therapy is not widely available in low-resource settings that bear the greatest burden of morbidity and mortality globally.

How can we provide oxygen therapy safely and effectively?

Safe and effective use of oxygen requires (i) prompt and accurate detection of hypoxaemia, and (ii) appropriate administration of oxygen[8]. This must be combined with appropriate clinical evaluation and management of the underlying condition.

Who is oxygen useful for?

Oxygen is useful for all children and adults with hypoxaemia – and we know that hypoxaemia is common in serious illnesses at all ages. A systematic review found that hypoxaemia was observed in 19.1% of 2464 neonates in 4 studies, 2.9-17.1% of children with malaria in 4 studies, 2.7-14.6% of children with meningitis in 3 studies, and 1.8-8.3% of children with malnutrition in 4 studies[9]. Oxygen is also essential for the treatment of common adult illnesses, emergencies and for emergency obstetric care[12].

Which children should receive oxygen therapy?

Pulse oximetry is the standard available method for detecting hypoxaemia and determining who should receive oxygen therapy. The normal range for arterial oxygen pulsed saturation (SpO2) at sea level is 97-100%, and this is lower in mountainous regions due to the lower partial pressure of oxygen in arterial blood at higher altitudes[8].

<u>The current WHO guidelines suggest that we should give oxygen therapy to any child with SpO2<90%.</u>

A Sp02 of 90% corresponds to the beginning of the steep slope on the haemoglobin-oxygen dissociation curve. This means that when the SpO2 drops below 90% it becomes increasingly difficult for cells to extract enough oxygen from the blood.

What if I don't have pulse oximetry?

If pulse oximetry is not available, any child with a respiratory illness and any of the following clinical signs should receive oxygen therapy: central cyanosis; nasal flaring; inability to drink or feed (due to respiratory distress); grunting with every breath; or depressed mental state (i.e. drowsy, lethargic).

What about in Emergency situations?

Children presenting with *emergency signs* (obstructed or absent breathing, severe respiratory distress, central cyanosis, signs of shock, coma, or convulsions) should be given oxygen to keep SpO2≥95%, <u>during the resuscitation stage</u>.

Are there any Special Cases where I should give oxygen therapy to children with SpO2>90%?

In some conditions it may be appropriate to give supplemental oxygen at higher SpO2, such as severe anaemia, heart failure, sepsis, brain injury and peri-operatively. These states involve either impaired blood oxygen supply from lungs to body tissues, or particular susceptibility of certain vital organs. These children may be identified by the emergency signs. In these cases, it may be reasonable to target SpO2≥94%.

What if the child or family is scared of oxygen therapy?

Fear of oxygen therapy can sometimes make patients and families unwilling to be given oxygen. This is usually due to the perception that people who receive oxygen subsequently die (which may be true as the sickest patients are often given oxygen)[13]. Some people may also be fearful that the oxygen will be too expensive and result in catastrophic health expenditure. These fears can result in oxygen being turned off by parents or by staff. These fears can be addressed by:

- Providing parents with information about hypoxaemia and oxygen therapy.
- Showing parents blood oxygen levels using pulse oximetry – and showing improvement after starting on oxygen therapy.
- Clearly communicating the costs associated with receiving oxygen therapy.

How much oxygen should I give?

Start children on 1-2L/minute. Then titrate the flow rate to maintain SpO2 in the following target range:

- For most children, aim to maintain SpO2>90%
- For small (<2000g) or pre-term (<38 weeks GA) neonates, the target SpO2 also includes an upper limit to prevent risks of oxygen toxicity in this vulnerable population (e.g. retinopathy, lung damage). Aim for 90-95% (NB: some guidelines will say 85-90% or 88-92%).
- For children with anaemia, heart failure, sepsis, brain injury and peri-operatively, it may be reasonable to target SpO2≥94%.

What monitoring should I do for children receiving oxygen therapy?

Routine monitoring for children on oxygen should include 3-4 hourly observations by nursing staff, including pulse oximetry if possible. This should include:

- Oxygen saturation, by pulse oximeter
- Position of nasal prongs (clean and reposition if necessary)
- Leaks in the oxygen delivery system

- Correct oxygen flow rate
- Airway obstructed by mucus (clear the nose with a moist wick or by gentle suction)
- Remove and clean the prongs at least twice a day.

If pulse oximetry is limited, every child should have a SpO2 checked and documented at least once per day. Additional monitoring is required for:

- Children who develop apnoea, worsening respiratory distress, altered conscious state, or other signs of clinical deterioration – check SpO2 promptly.
- Children with severe respiratory distress – check SpO2 at least 3-4 hourly.
- Children who have had their oxygen flow rate changed in any way (started, stopped, increased, decreased) – recheck one hour after changing.

When can I stop oxygen therapy?

Trials off oxygen should be attempted at least once daily if they are clinically stable and there are no emergency signs. This requires disconnection from oxygen therapy for 10-15 minutes and pulse oximetry monitoring.

Children should not be discharged until their SpO2 has been stable >90% for 24 hours, all emergency signs have resolved, and appropriate home treatment is organised.

Oxygen Equipment – delivery devices

What oxygen delivery device is best?

Oxygen should be administered via nasal prong cannulas for infants and children. Where nasal prongs are not available, nasal or nasopharyngeal catheters can be used. Facemasks and head-boxes are not recommended.

This choice is based on safety, tolerability, and effectiveness[14]. Nasal prongs are very safe, well tolerated, and provide consistent concentrations of oxygen at 30-35% at 1L/min in a 5kg child (up to 60% in neonates). Nasopharyngeal catheters (using 6 or 8 French nasogastric tubes) achieve higher oxygen concentration (45-60% at 1L/min) but require humidification (because they bypass the nasopharynx), special nursing skill for insertion and monitoring, and they can irritate the airway causing increased secretions. Nasal catheters use a similar method, but are inserted into the nasopharynx (not beyond). Nasal catheters achieve similar oxygen concentration to nasal prongs, require moderate nursing skills, and can irritate the airway causing nasal secretions. Face masks achieve only low oxygen concentration (25-30% at 1L/min), make feeding difficult, and have a risk of carbon dioxide retention. Head boxes (sometimes used for neonates) provide extremely variable oxygen concentration, make feeding difficult, and have the major risk of carbon dioxide retention.

The commonest problem with all delivery devices is obstruction from airway secretions. Therefore, nurses should regularly check the delivery devices and clean or change them as necessary.

Table 1 Comparison of different methods of oxygen delivery*

Method	Oxygen concentration achievable with 1 l/min in a 5 kg child	Danger of CO_2 accumulation	Equipment required
Nasopharyngeal catheter	45–60%	No	Nasogastric tubes, humidifier
Nasal catheter	35–40%	No	Nasogastric tubes
Nasal prongs	30–35% up to 60% in neonates[6]	No	Nasal prongs
Oropharyngeal catheter	45–60%	No	Nasogastric tube and humidifier
Face mask	29%	Yes	Face mask
Head box	Variable	Yes	Head box

Table 3 Summary of relative safety, tolerability and complications associated with low-flow oxygen delivery methods

	Nasopharyngeal catheter	Nasal catheter	Nasal prongs
Efficiency	+++	++	+
Tolerability by patient	++	++	++
Nursing demand	+++	++	+
Mucous production and risk of airways obstruction by mucus	+++	+++	+
Safety	+	++	+++
Reported major complications	Perforation of airways[8,16–19]; Gastric distension[7]	Nasal bleeding[12]	Tube breakage[7]

How do I use nasal prongs?

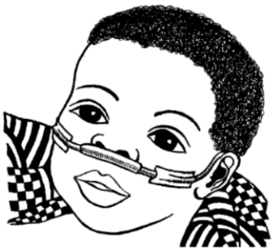

These are short tubes inserted into the nostrils. Place them just inside the nostrils, and secure with a piece of tape on the cheeks near the nose.

Care should be taken to keep the nostrils clear of mucus, which could block the flow of oxygen. Humidification is not required because the nasopharynx will provide it adequately.

How do I insert a nasal catheter?

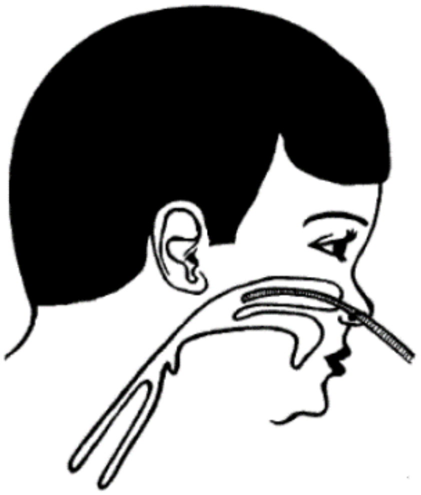

A 6 or 8 French gauge catheter that is passed to the back of the nasal cavity. Insert the catheter at a distance equal to that from the side of the nostril to the inner margin of the eyebrow.

Humidification is not required because the nasopharynx will provide it adequately.

How do I insert a nasopharyngeal catheter?

A 6 or 8 French gauge catheter is passed to the pharynx just below the level of the uvula. Insert the catheter at a distance equal to that from the side of the nostril to the front of the ear. If it is placed too far down, gagging and vomiting and, rarely, gastric distension can occur.

Humidification is required because it bypasses the nasopharynx.

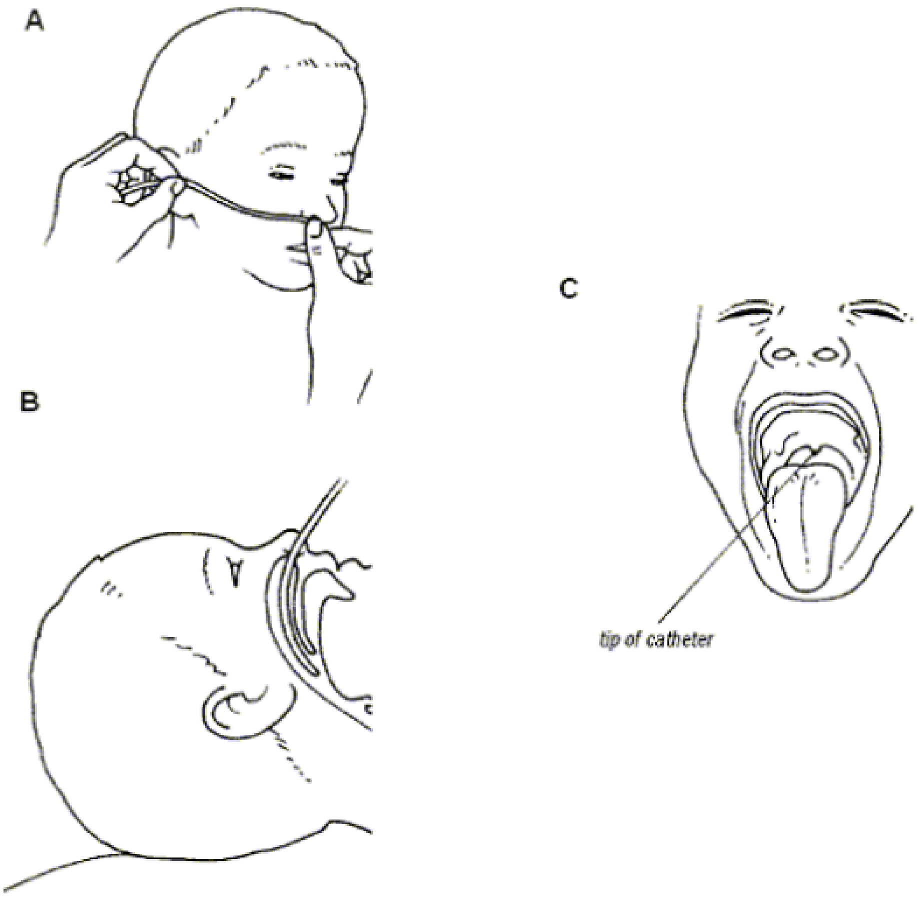

A: Measuring the distance from the nose to the tragus of the ear for the insertion of a N-P catheter
B: Cross-sectional view of the position of the N-P catheter
C: Tip of the N-P catheter visible just below the soft palate

How to I monitor oxygen delivery?

Train nurses to place and secure the nasal prongs correctly. Check regularly that the equipment is working properly, and remove and clean the prongs at least twice a day. Monitor the child at least every 3 h to identify and correct any problems, including:

- oxygen saturation, by pulse oximeter
- position of nasal prongs
- leaks in the oxygen delivery system
- correct oxygen flow rate
- airway obstructed by mucus (clear the nose with a moist wick or by gentle suction)

How can I be sure that oxygen is getting to the patient?

Clinically, users should look for improvement in the patient after starting oxygen. This typically includes: higher SpO2; lower respiratory rate; less respiratory distress; lower heart rate. However, it is also useful to check the gas flow and oxygen concentration regularly – especially if you are worried that the patient is not improving.

The simplest way to check that oxygen is getting to the patient is to perform the 'bubble test'. This involves immersing the nasal prongs in a beaker of clean water and testing at 0.5L/min and 1L/min. Users should see bubbles according to higher and lower flow rate. This will show you that gas is getting to the patient (but it doesn't tell you about the oxygen concentration). The bubble test should be done at least weekly as part of routine equipment checking.

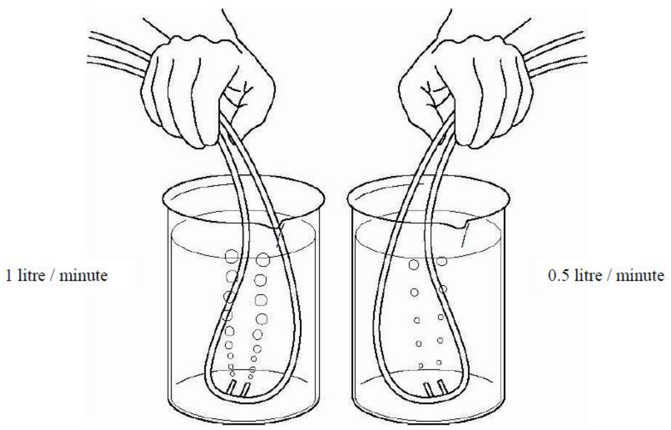

The oxygen concentration can be tested in two ways. Firstly, if you are using a concentrator it should have an alarm that comes on if the oxygen concentration being produced is <85%. Often this alarm will go on when the concentrator is first switched on, while the concentrator gets up to full capacity. Secondly, the oxygen concentration can be tested using oxygen analysers, which give an exact measurement of the amount of oxygen produced.

Can we share oxygen between multiple patients?

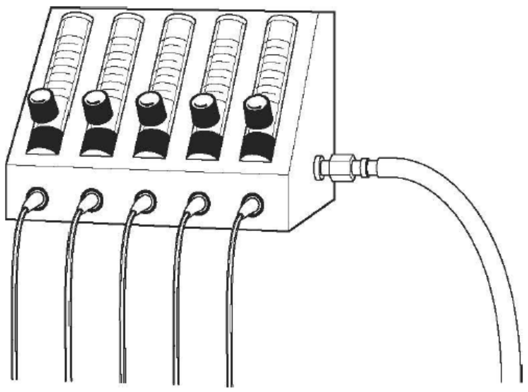

Sureflow devices allowing the sharing of one oxygen source between up to five patients. Users can individually titrate oxygen flow rates. The maximum cumulative flow rate is equal to the flow rate entering the device.

For example, if you are using a 5L/minute concentrator the maximum cumulative flow rate is 5L/minute. If you turned all five outlets up to the maximum, they would all flow at ~1L/minute.

Sureflow devices are typically installed on the wall near the nurses desk so that they can be easily checked and adjusted. They are numbered according to the bed that they supply.

What about "flow splitters"?

Flow splitters are older devices that can also enable one oxygen source to be shared between multiple patients. Users guide flow through flow splitters by blocking or unblocking the outlets. Users cannot individually titrate flow rates, and a common problem is loss of oxygen from outlets that have accidentally been left open

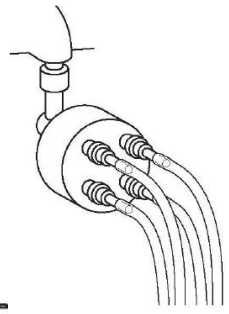

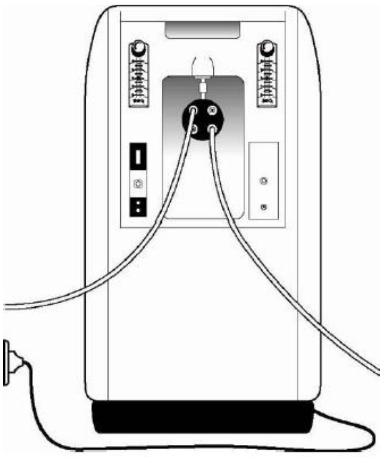

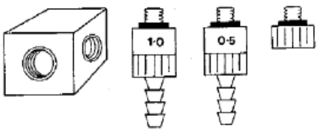

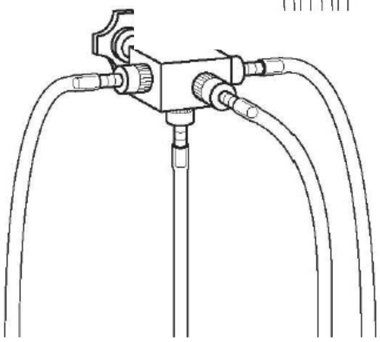

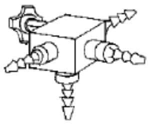

Oxygen equipment - Concentrators and other oxygen sources

What different sources of oxygen are there?

The three main sources of oxygen are: oxygen cylinder; oxygen concentrators; or oxygen generator. For individual health facilities, the choice of oxygen system will depend on (i) how much oxygen is likely to be needed, (ii) reliability of current electricity supply, (iii) existing oxygen infrastructure (e.g. oxygen piping into hospitals), (iv) geography and other access factors, and (v) public and commercial procurement options.

What about oxygen cylinders?

Oxygen cylinders have been the traditional mainstay of oxygen supply in low-resource settings. A G-size cylinder holds ~7000 litres of oxygen (filling pressure of 13000 kPa), and pressure regulators and flow-metres are needed to regulate flow from the cylinder to patients. Oxygen cylinders are typically expensive and for some countries this has resulted in oxygen being the single largest drug expense by the government (e.g. PNG[15]). The main costs are: purchase of gas; purchase/rent of cylinders; and transport. Costs are elevated by (i) high energy requirements of manufacturers to produce oxygen gas by low-temperature liquefaction distillation, (ii) high transport costs and associated loss during transit, (iii) private company monopolies on sale and rent, (iv)[15]. These factors are further exaggerated in areas that are geographically remote or otherwise difficult to access[15].

What about oxygen generators?

Oxygen generators are large oxygen concentrators intended for the supply of entire hospitals, and can generate the equivalent of 2-100 cylinders of oxygen per day (14,000-700,000L). They use the same technology as oxygen concentrators, and require reliable electricity supply. The oxygen produced can be piped into the hospital, or used to fill cylinders to store and transport around the hospital. Oxygen generators are custom-built and trained technicians are essential.

What about oxygen concentrators?

Oxygen concentrators are small machines that use gas chromatography technology to convert ambient air into 85-95% oxygen at 5-20 l/min[16]. With a flowmeter assembly, a single oxygen concentrator can deliver oxygen to up to 5 children simultaneously. The main costs are: equipment (and ongoing supplies); installation; and maintenance. Most maintenance can be done by appropriately trained local staff, with internal repairs conducted by trained technicians/engineers. The requirement of a reliable electricity supply (continuous 240V, 50Hz) can mean significant additional infrastructure costs to facilities where electricity supply is lacking or unreliable. Also, the inability of concentrators to produce sufficient flow for most anaesthetic machines means that specialised machines may be needed in hospitals that perform surgery.

How does an oxygen concentrator work?

Oxygen concentrators take in air (21% O2, 88% Nitrogen) and uses a zeolite filter to absorb nitrogen and produce ~95% oxygen. This is done using a pressure swing adsorption (PSA) process, where air flow is rapidly alternated across two zeolite filled columns which turns to absorb nitrogen and then release back into the air[16].

Concentrators also have internal and external filters to remove particles and environmental pathogens and ensure the air produced is clean. These filters need regularly cleaning to prevent overheating and concentrator failure – this is particularly critical in dusty environments.

How do I look after oxygen concentrators?

Oxygen concentrators can work well for more than 10 years if they are looked after well. This requires routine user maintenance (weekly), and preventive maintenance by trained technicians (6 monthly). Systems need to be established to ensure that this is done regularly, and recorded in logbooks.

Routine user maintenance can be done using this simple checklist (which also includes checking your pulse oximeters):

1. **Turn the Concentrator on. Does an audible alarm sound loudly for 5 second?** If the alarm is weak, or does not sound at all, contact the Engineer Responsible.
2. **Turn the Concentrator flowmeter to 5LM.**
3. **Does the Oxygen Concentration indicator (yellow light) turn OFF after a few minutes?** If it stays on, the concentrator is not producing adequate oxygen and needs referral to the Engineer Responsible.
4. **Check the tubing connections of the Concentrator and Flowmeter assembly.** Tighten loose connections, and if there are cracks or leaks inform the Engineer Responsible.
5. **Submerge the nasal prongs in water and adjust the flow rate on the Flowmeter Assembly** (Sureflow). If bubbles are not produced appropriately, contact the Engineer Responsible. Repeat for every flowmeter.
6. **Clean the concentrator body, check for damage, and look at the hours of use.**
7. **Remove the external filter and replace with a clean, dry filter.** Wash the old filter and allow to dry.
8. **Check the Lifebox Pulse Oximeter on your finger, and check each of the probes.** If any are not working, contact the Engineer Responsible.

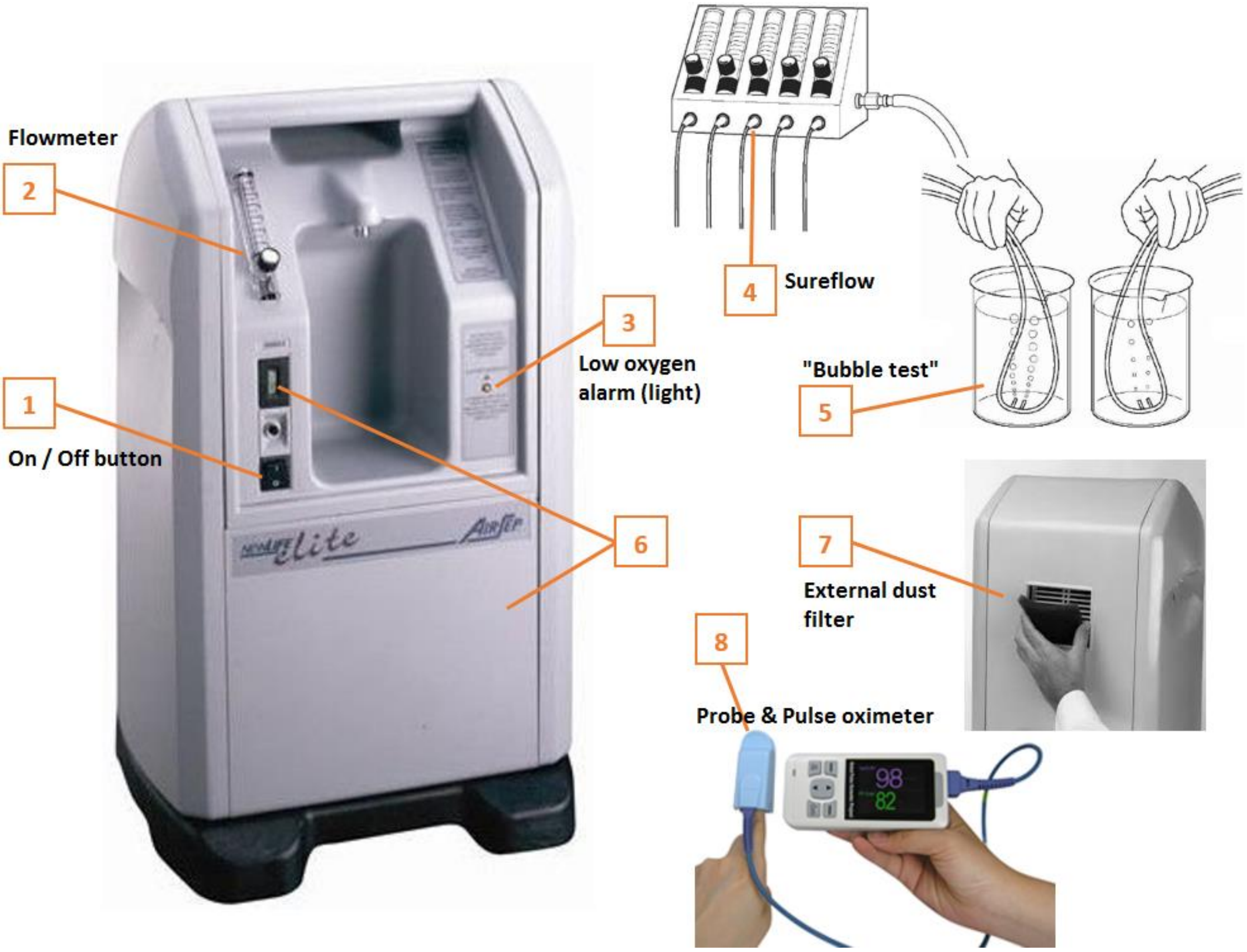

Pneumonia

What is the difference between diagnosing pneumonia and severe pneumonia (WHO definitions)?

Pneumonia involves cough or difficulty breathing PLUS one of the following:

- Fast breathing (age 2-11 months ≥50/min; age 1-5 years ≥40/min)
- Chest indrawing

Severe pneumonia involves cough or difficulty breathing PLUS one of the following:

- Hypoxaemia (SpO2 <90%) or Central Cyanosis
- Severe respiratory distress
  - Grunting
  - Severe chest-indrawing
- Danger signs*
  - Lethargy/reduced conscious state
  - Convulsions

Why is it important to distinguish between pneumonia and severe pneumonia?

Children with (simple) pneumonia can be treated at home with appropriate oral antibiotics. Parents should be told to return if the child becomes more unwell, and a health worker should review the child after 3 days to make sure they are getting better.

Children with severe pneumonia need admission to hospital. They need antibiotic treatment, and they often need additional treatment such as oxygen therapy, assistance with feeding, and treatment of other conditions.

The classification of pneumonia should be documented by the nurse or doctor who admits the child, so that everyone will know how sick they are and what treatment they need.

How do I assess a child for fast breathing?

Remove the child's shirt so that you can easily see the chest and abdomen. Watch the child's breathing. Count the breaths for 1 minute, using your watch.

Babies and infants can have very irregular breathing, so it is best to count over a full minute. (For older children, you may be able to count the breaths for 30 seconds, then multiply by two)

- Fast breathing for newborn babies (age <4 weeks) is ≥60 breaths per minute.
- Fast breathing for infants (age 2-11 months) is ≥50 breaths per minute.
- Fast breathing for children (age 1-5 years) is ≥40 breaths per minute.
- Fast breathing for older children (age 5-15 years) is ≥30 breaths per minute.
- Fast breathing for adults (age >15 years) is ≥24 breaths per minute.

How do I assess a child for chest indrawing?

In normal breathing, the chest wall expands outwards during inspiration (breathing in) and contracts inwards during expiration (breathing out). In difficult breathing, the chest wall may be sucked inwards during inspiration due to the extra effort. You can see this extra effort by sucking in at the bottom of the neck (tracheal tug), sucking in between the ribs (intercostal recession), and sucking in at the bottom of the ribcage (subcostal recession / lower chest wall indrawing).

Lower chest wall indrawing is the best sign to look for. Babies and very young children have very soft chest walls and some lower chest wall indrawing can be normal when they are crying or upset. However, in respiratory illnesses such as pneumonia, this will become more severe and may occur with every breath.

Remove the child's shirt so that you can easily see the chest and abdomen. Watch the child's breathing. Wait until the child is relaxed (not crying, not straining, not moving) and look for lower chest wall indrawing. Assess the severity. If it is sucking in severely, this is a sign of severe illness.

How do I assess for central cyanosis or hypoxaemia?

Hypoxaemia is very difficult to detect using clinical signs alone, and pulse oximetry is the best tool (see pulse oximetry FAQs).

Central cyanosis may become visible when the SpO2 is <80%. Look at the child's lips and tongue. A blue/purple colour indicates central cyanosis.

NB: blue/purple colour on the extremities (fingers, hands, feet, toes) is call peripheral cyanosis and it is most commonly due to restricted blood flow (not hypoxaemia).

How do I assess a child for grunting?

Sometimes children with severe respiratory illnesses make a soft grunting noise during expiration (breathing out). Watch the child's breathing, and listen for a noise during expiration. Occasional grunting in babies may be normal, but it is abnormal if it occurs with every breath.

How do I assess a child for lethargy or reduced conscious state?

A lethargic child does not respond to stimuli as they would normally.

A simple way to assess conscious state is using AVPU. First call them by name (without touching them). If they do not respond touch them. If there is still no response press on the base of their fingernail or rub over the sternum bone to see if they respond to pain.

- A = Alert and responsive
- V = responds to Voice
- P = responds to Pain
- U = completely Unresponsive

A more detailed assessment can be done using the Glasgow Coma Scale (GCS):

	1	2	3	4	5	6
Eye	Does not open eyes	Opens eyes in response to painful stimuli	Opens eyes in response to voice	Opens eyes spontaneously	N/A	N/A
Verbal	Makes no sounds	Incomprehensi ble sounds	Utters inappropriate words	Confused, disoriented	Oriented, converses normally	N/A
Motor	Makes no movements	Extension to painful stimuli (decerebrate response)	Abnormal flexion to painful stimuli (decorticate response)	Flexion / Withdrawal to painful stimuli	Localizes painful stimuli	Obeys commands

When can a child with severe pneumonia be discharged?

Children with severe pneumonia can be discharged when:

- Respiratory distress has resolved.
- There is no hypoxaemia (oxygen saturation, > 90%).
- They are feeding well.
- They are able to take oral medication or have completed a course of parenteral antibiotics.
- The parents understand the signs of pneumonia, risk factors and when to return.

DOES THIS PATIENT NEED OXYGEN?

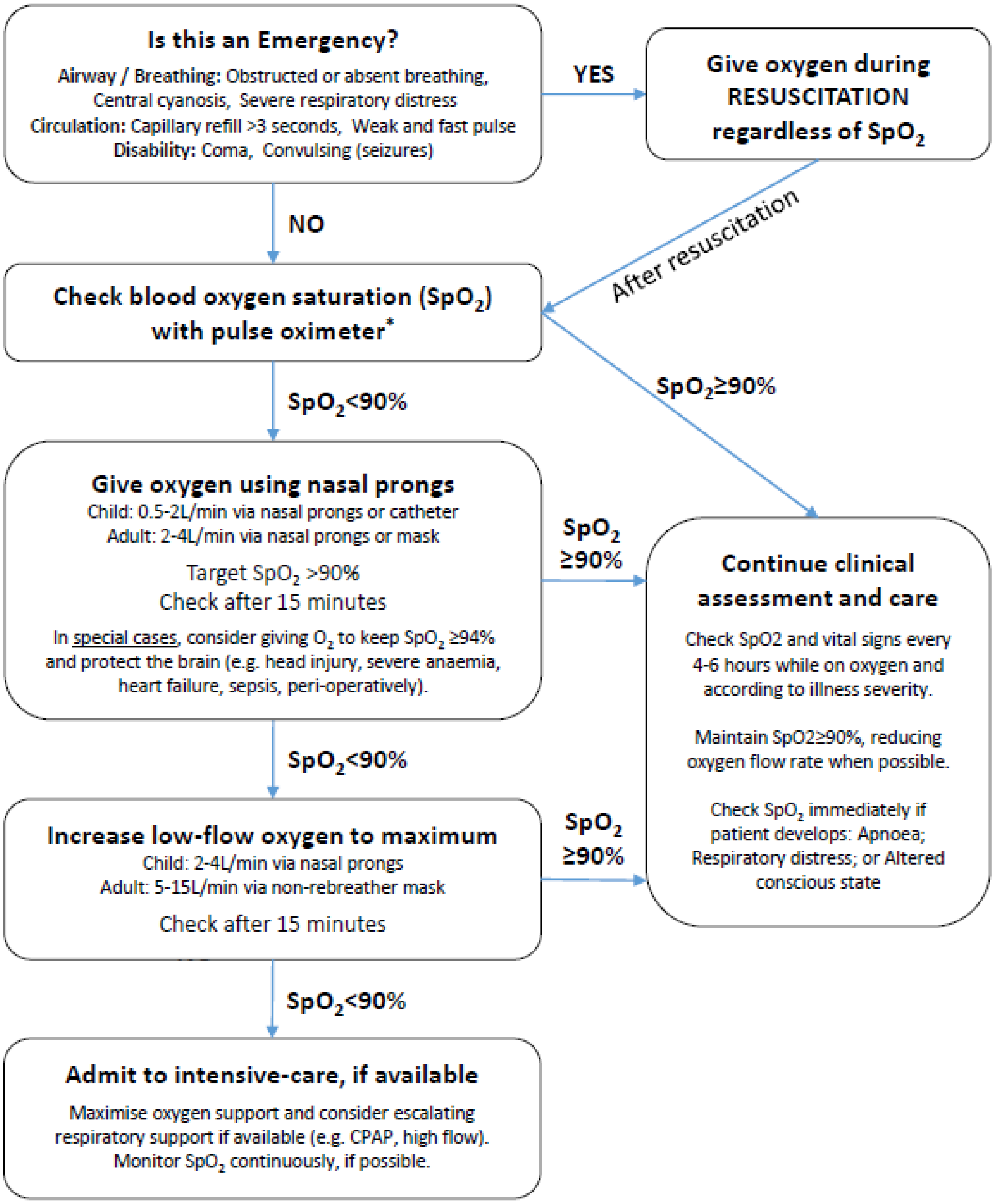

* If you do not have a pulse oximeter, use clinical signs:

central cyanosis, severe lower chest wall indrawing, RR≥70 breaths per minute, inability to drink (due to respiratory distress), grunting with every breath, depressed mental state.

MONITORING AND STOPPING OXYGEN THERAPY

Adults and Children (except preterm/small neonates)

Check blood oxygen saturation (SpO_2) using pulse oximetry

- Attach probe to a finger, toe, or ear
- Wait until there is a regular pulse signal (this may take 60 seconds)

Troubleshoot

- Reposition probe
- Check circulation (if poor, resuscitate immediately)
- Check oximeter function on own finger
- Warm the finger or toe

Read the SpO_2 - record SpO_2 and oxygen flowrate on a monitoring chart

What is the SpO_2?

SpO_2 <90%

TOO LOW => Increase O_2 therapy

Max flow-rate:
Child = 2-4L/min via nasal prongs
Adult = 5-15L/min via non-rebreather mask

Check the SpO_2 after 15 minutes, repeat in an hour

SpO_2 ≥90% → GOOD

SpO_2 <90%

Consider increasing respiratory support

Admit to intensive-care.

Maximise oxygen support and consider escalating respiratory support if available (e.g. high flow, CPAP, NIV/BiPAP).

Monitor SpO_2 continuously.

SpO_2 ≥90%

GOOD => Continue O_2 therapy

Continue routine monitoring

If SpO_2 ≥96%, try reducing (or ceasing) oxygen therapy daily

Check the SpO_2 after 15 minutes, repeat in an hour

Repeat as above

Routine SpO_2 monitoring

Monitor SpO_2 and vital signs at least 4 times daily in all patients, and 4-6 hourly while on oxygen

- Aim to keep SpO_2 between 90-95% at all times
- Monitor more frequently in patients with severe respiratory distress (preferably continuously)
- Recheck SpO_2 immediately if patient develops apnoea, worsening respiratory distress, altered conscious state, or other signs of clinical deterioration
- Consider oxygen therapy to maintain SpO_2 ≥95% in special cases (e.g. severe anaemia, shock)

Document SpO_2 and oxygen flow rate on a monitoring chart

Clean the nasal prongs each time you check the SpO_2 to ensure they are patent

MONITORING AND STOPPING OXYGEN THERAPY

Preterm (<37 weeks GA), Small (birth weight <2000g)

Check blood oxygen saturation (SpO_2) using pulse oximetry

- Attach probe to a finger, toe, or ear
- Wait until there is a regular pulse signal (this may take 60 seconds)

Troubleshoot

- Reposition probe
- Check circulation (if poor, resuscitate immediately)
- Check oximeter function on own finger
- Warm the finger or toe

Read the SpO_2 - record SpO_2 and oxygen flowrate on a monitoring chart

What is the SpO_2?

<90%

TOO LOW

<u>Increase</u> O_2 therapy

Max flow-rate = 2L/min via nasal prongs

Check the SpO_2 after 15 minutes, repeat in an hour.

SpO_2 <90%

Consider increasing respiratory support

Maximise oxygen support and consider escalating respiratory support if available.
(e.g. CPAP, high flow)

Monitor SpO_2 continuously.

90-95%

GOOD

<u>Continue</u> O_2 therapy

Continue routine monitoring

>95%

TOO HIGH

<u>Decrease</u> O_2 therapy

Check the SpO_2 after 15 minutes, repeat in an hour.

Repeat as above

Routine SpO_2 monitoring

Monitor SpO_2 and vital signs at least 4 times daily in all patients, and 4-6 hourly while on oxygen

- Aim to keep SpO_2 between 90-95% at all times
- Monitor more frequently in patients with severe respiratory distress (preferably continuously)
- Recheck SpO_2 immediately if patient develops apnoea, worsening respiratory distress, altered conscious state, or other signs of clinical deterioration
- Consider oxygen therapy to maintain SpO_2 ≥95% in special cases (e.g. severe anaemia, shock)

Document SpO_2 and oxygen flow rate on a monitoring chart

Clean the nasal prongs each time you check the SpO_2 to ensure they are patent

HOW TO PERFORM (AND TROUBLESHOOT) PULSE OXIMETRY

Prepare oximeter and patient

- Select appropriate probe for patient
- Connect probe to oximeter and turn oximeter on
- Tell the patient/parent what you are going to do
- Position the patient so they are comfortable (e.g. on parent's lap)

Attach probe to patient and wait

- Attach probe to a finger, toe, or ear
- Wait until there is a regular pulse signal (may take 20-30 seconds)

Is there a good Pulse Signal (waveform)?

NO → **Troubleshoot** (then recheck)

- Reposition probe
- Check circulation (if poor, resuscitate immediately)
- Check oximeter function on own finger
- Warm the finger or toe

YES →

Read the SpO2 - record SpO2 on a monitoring chart

What is the SpO_2?

≥90% →

Continue clinical assessment and care

Check SpO2 and vital signs every 4-6 hours while on oxygen and according to illness severity.

Maintain SpO2≥90%, reducing oxygen flow rate when possible.

Check SpO_2 immediately if patient develops: Apnoea; Respiratory distress; or Altered conscious state

Clean the nasal prongs each time you check the SpO2 to keep them patent

---

In Emergencies give oxygen during resuscitation regardless of SpO_2 : obstructed/absent breathing, central cyanosis, severe respiratory distress, capillary refill >3 seconds, weak & fast pulse, coma, seizures.

In Special Cases give oxygen to keep SpO_2 ≥94% and protect the brain: head injury, meningitis, severe anaemia, heart failure, sepsis, peri-operatively.

<90% →

Treat immediately if SpO_2 <90%

(assume HYPOXIA until proven otherwise)

- Give oxygen: Child = 0.5-2L/min via nasal prongs or catheter; Adult = 2-4L/min via nasal prongs or mask
- Recheck SpO_2: record SpO_2, O_2 flow rate

Recheck the SpO_2?

≥90% → Continue clinical assessment and care

<90% →

Increase O_2 therapy and recheck patient

(it is essential to correct hypoxia)

Turn oxygen flow rate up to increase SpO_2 ≥90%

- Maximum rate: 4L/min for a child, 2L/min for an infant, 5-15L/min for an adult (non-rebreather mask).

If remains low

- Check equipment (oximeter and oxygen supply)
- Check patient airway, suction nose
- Check circulation
- Consider medical causes (e.g. pneumothorax, congenital heart disease)
- Consider extra respiratory support (e.g. CPAP)

Weekly Equipment Checklist

To keep the Oxygen Concentrator, Pulse Oximeter, and other equipment functioning effectively please perform these basic checks every week, and record on the Checklist. If there are any problems, report them to the Engineer/Technician responsible within 24 hours.

1. **Turn the Concentrator on.** If the concentrator has no power supply, an alarm may sound. If the power failure alarm does not alarm, inform the responsible technician.
2. **Turn the Concentrator flowmeter to 5LPM (or maximum).**
3. **Use the Oxygen Concentration Indicator (OCI), or oxygen analyser, to test oxygen purity.** If the OCI stays on, the concentrator is not producing adequate oxygen. Contact the Engineer/Technician responsible.
4. **Check the tubing connections of the Concentrator and Flowmeter stand.** Tighten loose connections, and if there are cracks or leaks inform the Engineer Responsible.
5. **Submerge the nasal prongs in water and adjust the flow rate on the Flowmeter stand**. If bubbles are not produced appropriately at flow rates of 0.5-2.0 LPM, contact the Engineer/Technician responsible. Repeat for every flowmeter. (You can now turn the concentrator off)
6. **Clean the concentrator body, check for damage, and record the hours of use.**
7. **Remove the external filter and replace with a clean, dry filter.** Wash the old filter and allow to dry.
8. **Check the Pulse Oximeter on your finger, and check that each of the probes are working.** If any are not working, contact the Engineer/Technician responsible.

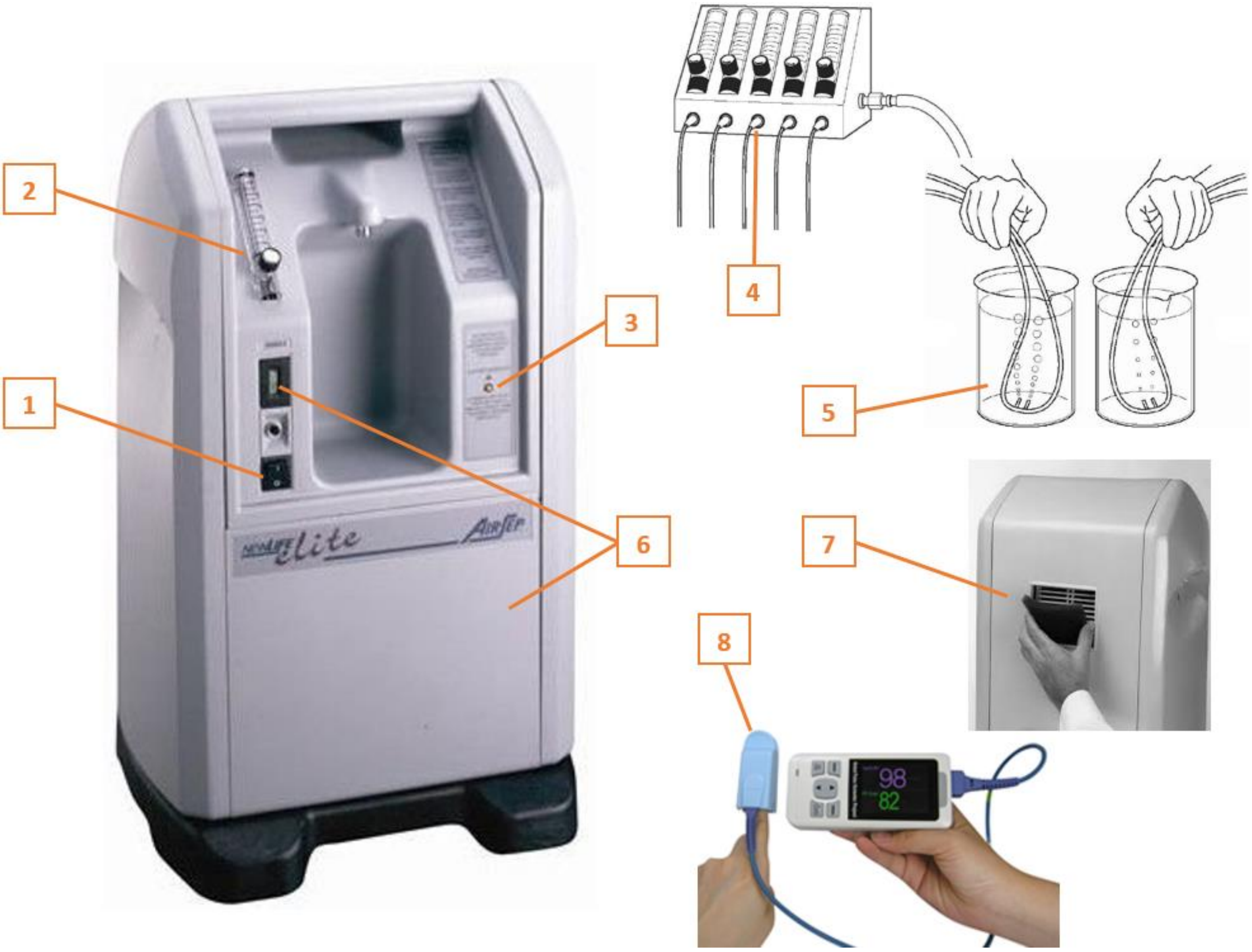

Weekly Equipment Checklist Hospital:____________________________

Ward:_________________ Quarter:____

Complete this EVERY WEEK. Check every item of equipment. Indicate correct function of all equipment with ✓, and any problems with ✗.

Describe problems identified and action taken (including equipment serial number).

		Concentrators				Oximeters		Document problems identified and action taken (include serial number)	
	**Checklist item ->**	①②③	④⑤	⑥	⑦	⑧			
**Week**	**Date**	**Alarms**	**Flow**	**Hours**	**Filter**	**Oximeter**	**Probes**	**Problems & Actions**	**Sign**
E.g.	*20/02/20*	✓	✓	4	✓	✓	✗	*Probe #1234567 broken, contacted Engineer for replacement.*	*HG*
1									
2									
3									
4									
5									
6									
7									
8									
9									
10									
11									
12									
13									

References

1. Piaget J. The role of action in the development of thinking. In: Overton WF, Gallagher JM, eds. Knowledge and Development: Plenum Press; 1977: 17--42.

2. Bandura A. Social foundations of thought and action: A social cognitive theory: Prentice-Hall, Inc; 1986.

3. Merrill MD. First Principles of Instruction. In: Reigeluth CM, Carr A, eds. Instructional Design Theories and Models: Building a Common Knowledge Base. New York: Routledge Publishers; 2009.

4. Merrill MD. Reflections on a four decade search for effective, efficient and engaging instruction. In: Allen MW, ed. Michael Allen's 2008 e-Learning Annual: Wiley Pfieffer; 2008: 141-67.

5. DeZure D, Kaplan M, Deerman MA. Reserach on student notetaking: implications for faculty and graduate student instructors. Center for Research on Learning and Teaching, University of Michigin: University of Michigin.

6. Friedman MC. Notes on Note-Taking: Review of Research and Insights for Students and Instructors. Harvard Initiative for Learning and Teaching: Harvard University.

7. Jordan LA, Papp R. PowerPoint: It's not "yes" or "no" - it's "when" and "how". *Research in Higher Education Journal* 2013; **22**(Dec 2013): 1-12.

8. Duke T, Subhi R, Peel D, Frey B. Pulse oximetry: technology to reduce child mortality in developing countries. *Ann Trop Paediatr* 2009; **29**: 165-75.

9. Subhi R, Adamson M, Campbell H, Weber M, Smith K, Duke T. The prevalence of hypoxaemia among ill children in developing countries: a systematic review. *Lancet Infect Dis* 2009; **9**: 219-27.

10. Ayieko P, English M. In children aged 2-59 months with pneumonia, which clinical signs best predict hypoxaemia? *Journal of Tropical Pediatrics* 2006; **52**: 307-10.

11. Duke T, Blaschke aJ, Sialis S, Bonkowsky JL. Hypoxaemia in acute respiratory and non-respiratory illnesses in neonates and children in a developing country. *Arch Dis Child* 2002; **86**: 108-12.

12. Duke T, Graham SM, Cherian MN, et al. Oxygen is an essential medicine: A call for international action. *International Journal of Tuberculosis and Lung Disease* 2010; **14**: 1362-8.

13. Stevenson AC, Edwards C, Langton J, Zamawe C, Kennedy N. Fear of oxygen therapy for children in malawi. *Arch Dis Child* 2015; **100**(3): 288-91.

14. Muhe L, Weber M. Oxygen delivery to children with hypoxaemia in small hospitals in developing countries. *International Journal of Tuberculosis and Lung Disease* 2001; **5**: 527-32.

15. Duke T, Peel D, Wandi F, Subhi R, Martin Sa, Matai S. Oxygen supplies for hospitals in Papua New Guinea: a comparison of the feasibility and cost-effectiveness of methods for different settings. *Papua and New Guinea medical journal* 2010; **53**: 126-38.

16. Duke T, Peel D, Graham S, Howie S, Enarson PM, Jacobson R. Oxygen concentrators: a practical guide for clinicians and technicians in developing countries. *Ann Trop Paediatr* 2010; **30**: 87-101.
